# An invasive beetle–fungus complex is maintained by fungal nutritional-compensation mediated by bacterial volatiles

**DOI:** 10.1038/s41396-020-00740-w

**Published:** 2020-08-19

**Authors:** Fanghua Liu, Jacob D. Wickham, Qingjie Cao, Min Lu, Jianghua Sun

**Affiliations:** 1 State Key Laboratory of Integrated Management of Pest Insects and Rodents, Institute of Zoology, Chinese Academy of Sciences, 100101 Beijing, China; 2 CAS Center for Excellence in Biotic Interactions, University of Chinese Academy of Sciences, 100049 Beijing, China; 3 College of Forestry, Hebei Agricultural University, 071000 Baoding, China; 4 Present Address: State Key Laboratory of Biology of Plant Diseases and Insect Pest, Institute of Plant Protection, Chinese Academy of Agricultural Sciences, 100193 Beijing, China

**Keywords:** Microbial ecology, Microbial ecology

## Abstract

Mutualisms between symbiotic microbes and animals have been well documented, and nutritional relationships provide the foundation for maintaining beneficial associations. The well-studied mutualism between bark beetles and their fungi has become a classic model system in the study of symbioses. Despite the nutritional competition between bark beetles and beneficial fungi in the same niche due to poor nutritional feeding substrates, bark beetles still maintain mutualistic associations with beneficial fungi over time. The mechanism behind this phenomenon, however, remains largely unknown. Here, we demonstrated the bark beetle *Dendroctonus valens* LeConte relies on the symbiotic bacterial volatile ammonia, as a nitrogen source, to regulate carbohydrate metabolism of its mutualistic fungus *Leptographium procerum* to alleviate nutritional competition, thereby maintaining the stability of the bark beetle–fungus mutualism. Ammonia significantly reduces competition of *L. procerum* for carbon resources for *D. valens* larval growth and increases fungal growth. Using stable isotope analysis, we show the fungus breakdown of phloem starch into d-glucose by switching on amylase genes only in the presence of ammonia. Deletion of amylase genes interferes with the conversion of starch to glucose. The acceleration of carbohydrate consumption and the conversion of starch into glucose benefit this invasive beetle–fungus complex. The nutrient consumption–compensation strategy mediated by tripartite beetle–fungus–bacterium aids the maintenance of this invasive mutualism under limited nutritional conditions, exacerbating its invasiveness with this competitive nutritional edge.

## Introduction

Mutualistic associations between symbiotic microbes and animals are pervasive in every ecosystem [1–5], where they commonly compete with intraspecific and interspecific species within the host-provided environment [6–8]. These competitors include vector insects, other insects and coexisting microorganisms. Some specialist insects feed on nutrient-deficient food that experience particularly severe competition with their symbiotic microbes [9, 10]. Bark beetles (Coleoptera: Curculionidae: Scolytinae) are major economic and ecological pests in forest ecosystems that feed within nutritionally poor substrates including woody tissues, bark, phloem, and the pith of twigs [11, 12]. Most bark beetles vector symbiotic fungi that coexist with a multitude of microbes [12–16]. Bark beetle–fungi interactions have been classified as mutualistic, antagonistic, and commensal [12, 14]. Mutualistic fungi are often pathogenic to the host plant, facilitating their vector’s survival in living or newly killed plant tissues until the defenses subside [10]. Some mutualistic fungi also can induce the release of attractive volatiles from infected host trees, and these volatiles can facilitate aggregation of bark beetles on host trees [17]. Bark beetles associated with mutualistic fungi grow faster, have larger body size and their progeny suffer lower mortality [9, 18–21]. Meanwhile, fungi benefit from the mutualistic association by being vectored to otherwise inaccessible plant resources [19, 22]. However, the phloem that they consume has a low nutritional value relative to their requirements [21, 22], and it remains elusive exactly how these insects maintain mutualistic associations with beneficial symbionts under nutritional stress.

Nutritional contribution of beneficial symbionts has a critical role in maintaining the stability of the mutualism [9, 21, 23–26]. One well-studied insect-beneficial symbiosis is the interaction between aphids and their primary symbiont, *Buchnera*, which provides essential amino acids for its insect host [23, 27]. Similarly, genomic analysis of the obligate flavobacterial endosymbiont, *Blattabacterium* of *Periplaneta americana* also revealed that *Blattabacterium* were able to synthesize and provide all essential amino acids for *P. americana* [28]. In mosquitoes, gut microbiota mediate lipid absorption via the hypoxia pathway [25]. However, while these previous studies have extensively studied endosymbiotic and intestinal bacteria, little is known about the role of external microbes in contributing to nutrition. For nutritional deficiency in beetle–fungus symbioses, some symbiotic fungi serve as important food resources for the developing larvae to reduce nutritional challenges for beetles [19, 29]. In addition, some symbiotic fungi may expand the capacity of beetles to use nutrient-poor plant resources. For example, most plant tissues have relatively low levels of nitrogen and sterols, and the beetles’ mutualistic fungi benefit larval growth by concentrating nitrogen or producing sterols in phloem tissue where feeding occurs [9, 30]. For carbohydrates, however, mutualistic fungi compete with beetles for this resource leading to reduced larval growth [31]. In addition, antagonistic fungi also compete for the same carbohydrates, which reduced the stability of bark beetle–fungus mutualisms [10, 31, 32].

Carbohydrates are essential nutrients that provide energy and precursors for tissue building during bark beetle and fungi development [33]. Competition for this resource inevitably affects the stability of beetle–fungus mutualisms. Symbiotic bacteria of insects have been reported to have a stabilizing effect on the maintenance of insect–fungus mutualisms [1, 2], e.g., bark beetle-associated bacteria limited the growth and reproduction of symbiotic fungi [34–36]. Symbiotic actinomycetous bacteria can protect the beetle–fungus mutualism by producing antibiotics, which selectively inhibit antagonistic fungi [2]. However, only a few studies have focused on the effects of symbiotic bacteria on the bark beetle–fungus mutualism from the perspective of nutrition. The bacteria-released volatile, ammonia, has been found to be a regulator of the beetle–fungus mutualism and exposure to ammonia affected the symbiotic fungus’ growth and monosaccharides consumption, thereby contributing to the maintenance of the mutualistic community [37]. However, the underlying mechanisms behind how the volatile ammonia, which is produced by the beetle’s symbiotic bacteria, actually help them preserve the persistence of beneficial fungi through regulation of the fungus’ carbohydrate consumption, remains largely unknown. Previous studies show transcriptional regulators appeared to integrate control of both carbon and nitrogen metabolism in filamentous fungi [38–40].

Red turpentine beetle (RTB), *Dendroctonus valens* LeConte, native to North America, is a destructive invasive pest in China. It feeds on nutrient-poor phloem of the pine tree *Pinus tabuliformis Carrière*, causing extensive pine mortality in China [41]. The beetles are commonly associated with various ophiostomatoid fungi including *Leptographium procerum* and *Ophiostoma minus*, which coexist with a multitude of microbes [42, 43]. These fungal species were found in pits and on setae on their body surfaces and frass [42]. *L. procerum* is the most frequently isolated species from RTB and forms a tightly mutualistic beetle–fungus invasive complex, thereby contributing to RTB’s successful invasiveness in China [17, 42, 44]. However, this invasive complex faces a particularly severe challenge because the phloem that they consume is nutritionally poor (containing nitrogen-limited sources, recalcitrant carbohydrate sources, and plant defensive compounds) [9, 37]. Nitrogen concentrations (% dry weight) of *P. tabuliformis* and *Pinus taeda* phloem are 0.66% and 0.4%, respectively, whereas the nitrogen concentrations of fungal and insect tissues are in the ranges 1.5–7% and 8–13%, respectively [9, 37]. Bacteria associated with beetles cope with nitrogen limitation by recycling nitrogen from beetle excretions or fixing atmospheric nitrogen (N_2_) [9, 45, 46]. Carbon is in fact not limited but bound to polysaccharides in cellulose and starch, while easily accessible monosaccharides for RTB and its associated microbes are limited [31, 37]. RTB larval growth is drastically inhibited by competition over saccharides from *L. procerum* and some other fungi [31]. Further investigations revealed that three species of bacteria associated with RTB larvae (*Serratia liquefaciens* B310, *Rahnella aquatilis* B301, and *Pseudomonas* sp. 7 B321) produced the bacterial volatile ammonia, and experimentally adding ammonium eliminated the negative effects caused by symbiotic fungi on RTB larval growth [37]. In addition, associated bacterial volatiles inhibited the growth of antagonistic fungi *O. minus*, but had no effect on the growth of mutualistic fungi *L. procerum* by regulating monosaccharides consumption [37, 47]. All of these results demonstrated that the nitrogenous volatile ammonia released by several mutualistic bacterial strains apparently alleviate the nutritional competition for monosaccharides (i.e., d-glucose) between larvae and fungi [31, 37]. In this study, in order to elucidate the underlying mechanisms upon which a cryptic insect maintain mutualistic associations with beneficial symbionts under poor nutritional conditions, we examined the *D. valens*–*L. procerum* mutualism as a model. Results showed that the associated bacterial volatile ammonia, as a nitrogen source, regulates the mutualistic fungus *L. procerum*’s monosaccharide consumption and starch metabolism pathway that optimizes both the mutualistic fungus’ growth and larval development of RTB. We speculated that this is a strategy that helps maintain mutualistic associations with beneficial symbionts for insects in substrates with limited nutrition.

## Materials and methods

### Fungal strains and maintenance

The fungal strain *L. procerum* CMW25626, commonly associated with RTB and reported as a mutualistic fungal associate for RTB larvae, was used [17, 42]. The strain was cultured as previously described [31]. *Escherichia coli* DH5a bacterial strain was used for plasmid construction. *Agrobactium tumefaciens* AGL1 strain was used for fungal transformation [48].

### Ammonia, ammonium, and fungal growth in the phloem medium

Cultures of *L. procerum* were grown and maintained on 2% malt extract agar medium (MEA: 2 g malt extract, 2 g agar, and 100 mL purified distilled water). Phloem medium was prepared to examine the effects of ammonia and ammonium on the growth rate, dry weight, and density of *L. procerum*. The ammonia concentration measured in bacterial Luria–Bertani (LB) cultures and the effects of the serial dilution of ammonia water solution on growth and carbon source usage in *L. procerum* indicated that the regulation of ammonia and ammonium is concentration-dependent [37]. Medium preparation and experiments were performed as described in the Supplementary Methods.

### Ammonia, ammonium, and *D. valens* larval growth in the phloem medium

The effects of ammonia and ammonium on larval growth by *L. procerum* were determined using a phloem-medium method. The phloem medium was poured into petri dishes as described above. Experiments were performed as described in the Supplementary Methods.

### Effects of ammonium on carbohydrate composition in desugared phloem medium after *L. procerum* growth at different time points

In this experiment, in order to eliminate the effects of other carbohydrates and to show that the system is indeed carbon-limited in nature, we used desugared phloem medium, instead of standard phloem medium, to study the effects of ammonium on specific carbohydrate composition, then analyzed *L. procerum* growth at different time points. Medium preparation and experiments were performed as described in the Supplementary Methods.

### ^13^C_6_-labeled glucose treatment

In order to investigate the dynamics of glucose in the medium, a stable isotope of glucose (d-glucose-^13^C_6_) was utilized, and its concentration in the medium was measured by GC/MS. A total of 200 mg of d-glucose and d-glucose-^13^C_6_ standard aqueous solution (2 g/mL) was sampled and analyzed to obtain mass spectra with GC/MS [37]. To further clarify the source of the remaining glucose in the medium, d-glucose-^13^C_6_ 1 g/L and d-pinitol 1 g/L were added to desugared phloem medium. Particularly, the medium with ammonium was set as treatment and medium without ammonium was set as control. The desugared phloem medium with fungus was sampled on day 5 after the inoculation of fungus, and carbohydrate composition was measured [37]. An additional treatment with only d-pinitol 1 g/L added to desugared phloem was also analyzed using the same methods. For each treatment, eight biological replicates were used.

### Carbohydrate analysis

The carbohydrate content in phloem related to glucogenesis was extracted and analyzed. The starch was ground up determined using EnzyChrom Starch Assay Kit (BioAssay Systems, USA) according to the manufacturer’s instructions. For the starch extraction, we ground up 5 mg phloem, phloem medium and desugared phloem sample, and washed off any free glucose and small oligosaccharides with 1 mL 90% ethanol, then warmed it to 60 °C for extraction and analysis. Other polymers like pectin, xylan, and lignin that do not break up in d-glucose were not analyzed. The other carbohydrates, including sucrose, maltose, and trehalose, were also extracted and analyzed [37]. Eight replicates were used.

### RNA-seq analyses and qRT-PCR analysis

RNA extractions, RNA-seq analyses, cDNA synthesis, and qRT-PCR were performed as described in the Supplementary Methods. Three biological replicates were used for both RNA-sequence and qRT-PCR. The primers used here are included in Supplementary Table S1.

### Enzymatic activity

The enzymatic activities of the α-amylase, amyloglucosidase, and α-glucosidase of the fungus were measured using the corresponding model substrates (three replicates per treatment). Enzymes were extracted from fungi from three petri dishes in a centrifuge tube containing 1 mL of ice-cold PBS buffer (0.1 mol^−1^, pH = 7.4) [49]. The homogenate was centrifuged at 3500 rpm for 20 min at 4 °C and the resulting supernatant was used directly for spectrophotometric assays of AMYA, AMYG, and α-GLU activities. The activities of α-amylase, amyloglucosidase were determined using commercial ELISA (enzyme-linked immunosorbent assay) kits (Jiangsu Yutong Biological Technology Co., Ltd, Jiangsu, China), and α-glucosidase was measured by a commercial ELISA kit (Shanghai MLBIO Biotechnology Co., Ltd, Shanghai, China).

### Confirmation of starch as carbon source for d-glucose using minimal medium

The standard minimal medium was made as follows: 1 g of KH_2_PO_4_, 0.5 g of MgSO_4_·7H_2_O, 0.02 g of FeSO_4_·7H_2_O, 0.02 g of ZnCl_2_, 0.01 g of MnCl_2_, 0.01 g of Pyridoxine, and 25 g of Difco Bacto agar were dissolved into 1 L of distilled water. To further confirm the conversion of starch to glucose, 1 g of d-pinitol was added into the standard minimal medium. In addition to d-pinitol, either 20 g of starch, 20 g of cellulose, or 1 g of sucrose was also individually added to rule out their possible conversion to glucose. Eight biological replicates were used. For investigating the effects of d-glucose and starch on the growth of *L. procerum* and RTB larvae, 0.4% (w/v) of starch or d-glucose was added into standard minimal medium, respectively. pH of these media was adjusted to 6.0 ± 0.1 with 1 N KOH or 1 N H_2_SO_4_ before autoclaving (30 min, 120 °C, 0.14 Mpa). To ensure enough valid biological replicates, *n* = 32 was used.

### Gene knockout in *L. procerum*

Gene deletions of SUC1, AMYG, and AMYA3 were performed by homologous recombination [50, 51], as described in the Supplementary Methods. The primers used here are included in Supplementary Table S1.

## Results

### Ammonia, ammonium, and fungal growth

**Fig. 1:**
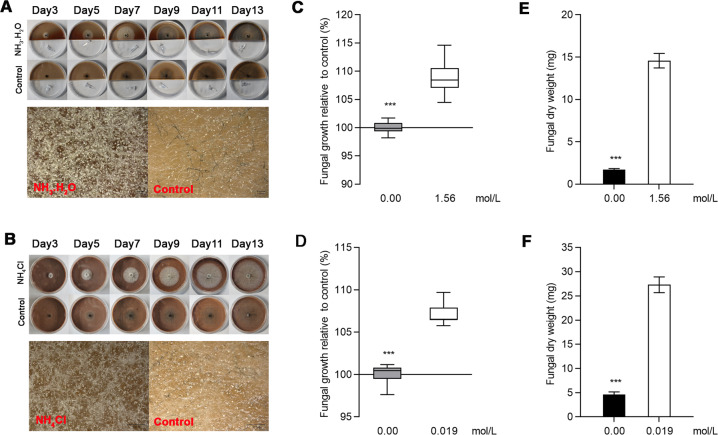
Exposure of ammonia and ammonium enhance the growth of *L. procerum*. Representative growth of *L. procerum* on phloem medium infused with ammonia (**a**) and ammonium (**b**), respectively (Top: Low-magnification images; Bottom: High-magnification images of samples from day 13). Effects of exposure of ammonia (**c**) and ammonium (**d**) on the growth of *L. procerum*. Effects of exposure of ammonia (**e**) and ammonium (**f**) on the dry weight of *L. procerum*. Error bars of **c**–**f** represent SEs of at least eight biological replicates. Asterisk indicates significant difference between treatment and control (*P < 0.05, **P < 0.01, ***P < 0.001). NS means no significance.

Laboratory-based experiments compared the presence or absence of ammonia/ammonium within the phloem medium with the growth performance of *L. procerum*. Ammonia/ammonium significantly promoted the growth of *L. procerum*. The spore and mycelium densities of *L. procerum* revealed that ammonia/ammonium exposure significantly increased the growth of *L. procerum* (Fig. 1a, b). The fungal growth rate was 109.0%, and 107.0% compared with the control at doses of 1.56 mol/L ammonia and 0.019 mol/L ammonium, respectively (Fig. 1c, d) (df = 35.241, *t* = −17.330, *P* < 0.001; df = 38, *t* = −12.403, *P* < 0.001 *t*-test). The activity on fungal growth by ammonia (1.56 mol/L) and ammonium (0.019 mol/L) was determined using the fungal biomass yield method. After 13 days, the fungal biomass yield with ammonia/ammonium (14.58 mg [dry weight] petri^−1^/27.30 mg [dry weight] petri^−1^) was significantly greater than that with sterile water (1.73 mg [dry weight] petri^−1^/4.60 mg [dry weight] petri^−1^) (Fig. 1e, f) (df = 7.223, *t* = 14.748, *P* < 0.001; df = 9.32, *t* = 13.213, *P* < 0.001 *t*-test).

### Ammonia, ammonium, and *D. valens* larval growth

**Fig. 2:**
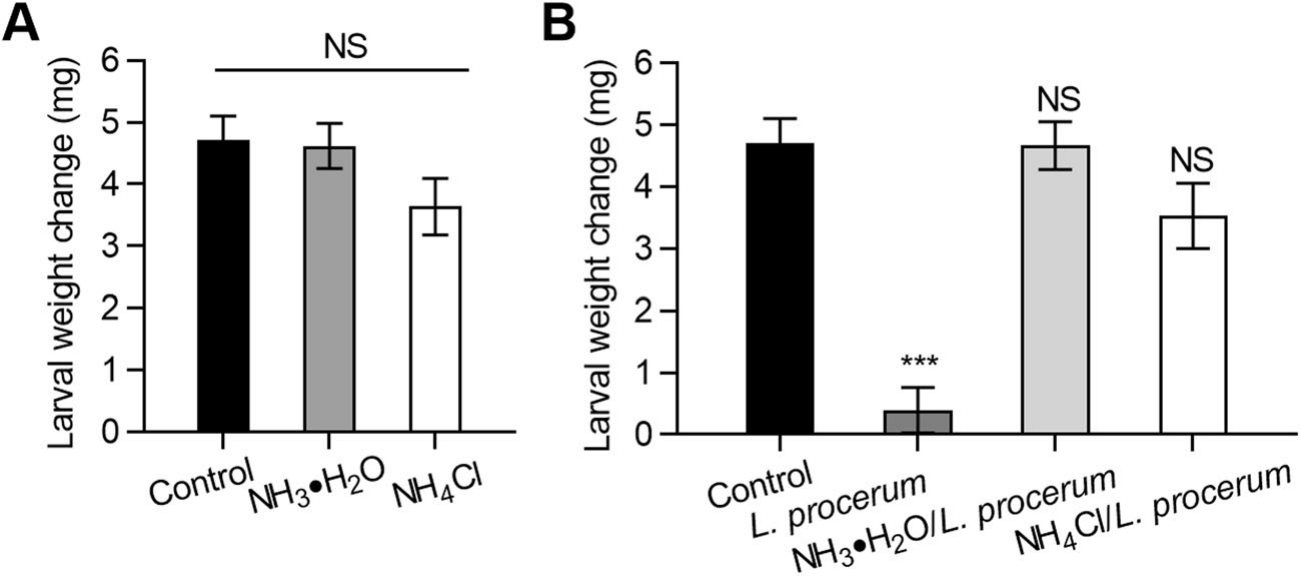
Exposure of ammonia and ammonium are beneficial for the association of RTB and *L. procerum*. (**a**) Effects of exposure of ammonia and ammonium on the weight change of RTB. (**b**) Effects of exposure of ammonia and ammonium on the weight change of RTB with the present of *L. procerum*. Error bars represent SEs of at least 20 biological replicates. Asterisk indicates significant difference between treatment and control (*P < 0.05, **P < 0.01, ***P < 0.001). NS means no significance.

We observed no significant difference in weight change of RTB larvae in phloem media with or without ammonia (1.56 mol/L)/ammonium (0.019 mol/L) (Fig. 2a) (*F*_2, 66_ = 2.163, *P* = 0.124 ANOVA, Dunnett’s test). Bioassays using the phloem medium infused with 1.56 mol/L ammonia or 1 g/L ammonium characterized the effects of ammonia and ammonium on RTB larval growth by *L. procerum*. RTB larval weight decreased significantly on *L. procerum*-colonized phloem media compared to fungus-free phloem media. The weight of RTB larvae fed with phloem media colonized by *L. procerum* with ammonia (1.56 mol/L)/ammonium (0.019 mol/L) presented were significantly higher compared to those with sterile water. In addition, no significant difference was noted between ammonia and ammonium treatments (Fig. 2b) (*F*_3, 80_ = 24.865, *P* < 0.0001 ANOVA, Dunnett’s test).

### Ammonium and carbohydrate consumption

**Fig. 3:**
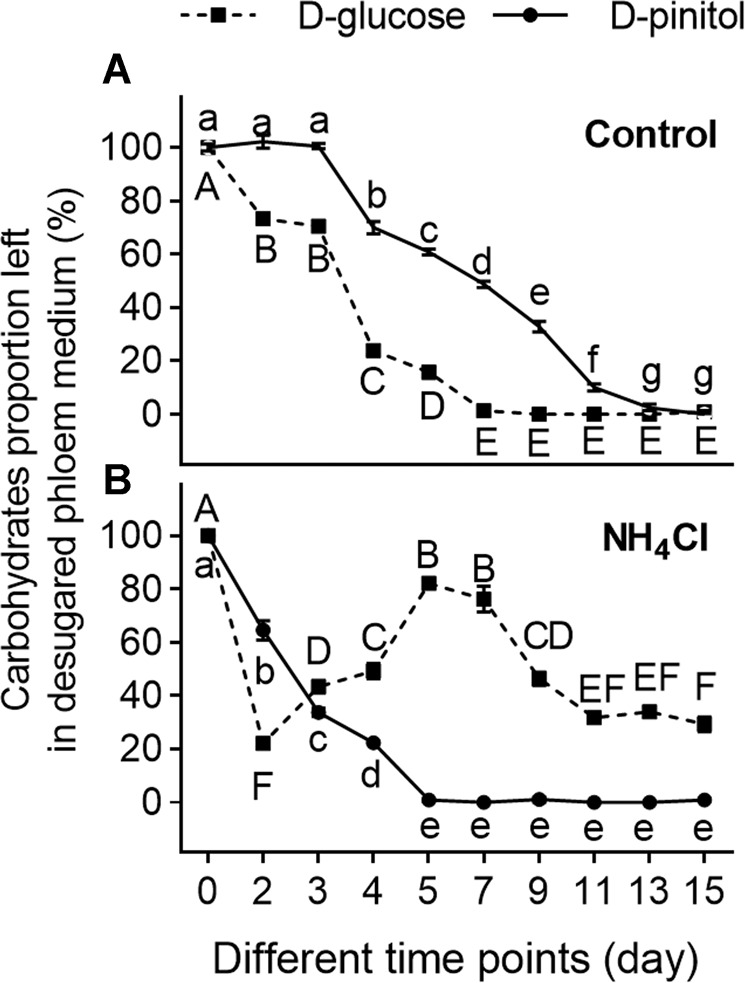
Exposure of ammonium alters the carbohydrate composition in *L. procerum*-colonized area of desugared phloem medium. Carbohydrate composition in *L. procerum*-colonized area of desugared phloem media at different time points without (**a**) and with (**b**) ammonium. Error bars represent SEs of at least eight biological replicates. Different letters indicate significant differences among different treatments (*P* < 0.05). NS means no significance.

Previously, we determined that ammonia can regulate *L. procerum* consumption sequence of d-pinitol and d-glucose in phloem medium but it did not affect the consumption of other carbohydrates [37]. Here, we verified the same result that ammonium can regulate the *L. procerum* consumption of d-pinitol and d-glucose using desugared phloem medium (adding 1 g/L of d-glucose and d-pinitol) (Supplementary Fig. S1) [37]. Compared to the untreated control cultures, the marked reduction in d-pinitol began earlier than d-glucose. d-glucose started to decline significantly on the 5th day in plates colonized by *L. procerum* without the ammonium, and was depleted on the 11th day. However, the content of d-pinitol stayed constant before the 7th day and 38.65% remained on the 11th day (Supplementary Fig. S1a) (d-pinitol: *F*_9, 70_ = 462.667, *P* < 0.001 ANOVA, Tukey’s test; d-glucose: *F*_9, 70_ = 1057.377, *P* < 0.001 ANOVA, Dunnett’s T3 test). In presence of ammonium, d-pinitol in plates colonized by *L. procerum* started to decline significantly on the 5th day and was used up on the 11th day, and 51.25% d-glucose remained in plates on the 11th day (Supplementary Fig. S1b) (d-pinitol: *F*_9, 70_ = 652.753, *P* < 0.001; d-glucose: *F*_9, 70_ = 97.468, *P* < 0.001 ANOVA, Dunnett’s T3 test). Ammonia and ammonium reduced inhibition of *L. procerum* on RTB larval growth and increased fungal growth by regulating d-glucose and d-pinitol consumption in the fungus (Figs. 1 and 2). Obviously, d-glucose was a readily available and preferred carbon source for *L. procerum* compared to d-pinitol [45]. In order to further confirm the relationship between the carbohydrates consumption by the fungus and the fungal growth in the presence of ammonium, we analyzed the carbohydrate composition of the desugared phloem medium (adding 1 g/L, d-glucose and d-pinitol) in *L. procerum*-colonized area as the fungus grew over time. Without the ammonium, the d-glucose and d-pinitol consumption by the fungus was similar to the whole desugared phloem medium and fungi-colonized area of the desugared phloem medium (Fig. 3a) (d-pinitol: *F*_9, 70_ = 628.211, *P* < 0.001; d-glucose: *F*_9, 70_ = 760.623, *P* < 0.001 ANOVA, Dunnett’s T3 test). With the presence of ammonium, d-pinitol also decreased significantly over time in plates colonized by *L. procerum*. On the other hand, the content of d-glucose in *L. procerum-*colonized area initially decreased sharply and then increased to its peak on day 5, and then decreased with time (Fig. 3b) (d-pinitol: *F*_9, 70_ = 564.548, *P* < 0.001 ANOVA, Dunnett’s T3 test; d-glucose: *F*_9, 70_ = 103.886, *P* < 0.001 ANOVA, Tukey’s test). Given these findings, we initially speculated that ammonia and ammonium do not affect d-glucose consumption by *L. procerum* and d-glucose left in the medium that originated from conversion from other substances.

### ^13^C_6_-labeled glucose treatment

**Fig. 4:**
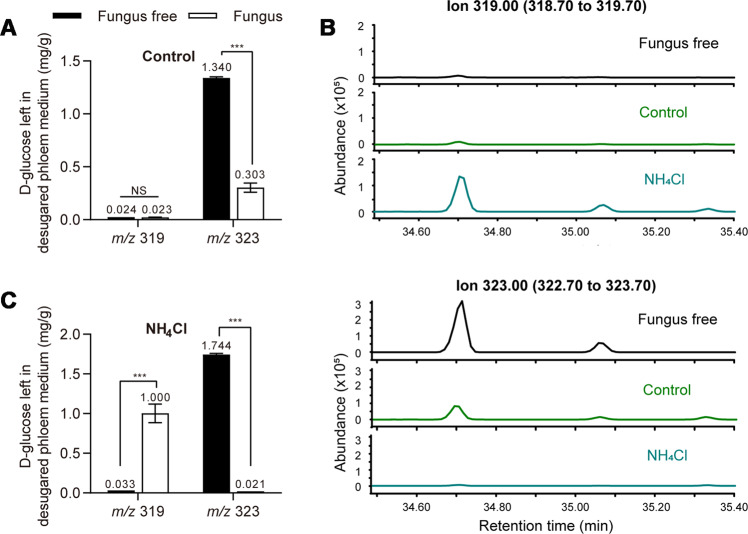
d-Glucose left in the medium is converted from some substance. Carbohydrate composition in *L. procerum-*colonized area of desugared phloem media at day 5 without (**a**) and with (**c**) ammonium. (**b**) GC–MS traces of natural glucose and ^13^C_6_-labeled glucose. Error bars of (**a**) and (**c**) represent SEs of eight biological replicates. Asterisk indicates significant difference between fungus and fungus-free (*P < 0.05, **P < 0.01, ***P < 0.001). NS means no significance.

The mass spectra of d-glucose and d-glucose-^13^C_6_ standards were shown in Supplementary Fig. S2. On the basis of the mass spectra for d-glucose and d-glucose-^13^C_6_, we decided to consider the ions *m*/*z* 319 and *m*/*z* 323, which have large mass numbers and relatively high intensities, as monitor ions. Without ammonium, there was no difference in the concentration of natural glucose between fungus-free and the fungus-colonized medium, and the concentration of labeled glucose significantly decreased in fungus-colonized medium when compared to the fungus-free medium (Fig. 4a, b) (*m*/*z* 319: df = 14, *t* = 1.110, *P* = 0.293; *m*/*z* 323: df = 5.686, *t* = 22.951, *P* < 0.001 *t*-test). In the presence of ammonium, the concentration of natural glucose increased and the labeled glucose was depleted on the fungus-colonized medium compared to the fungus-free medium (Fig. 4b, c) (*m*/*z* 319: df = 11, *t* = −8.309, *P* < 0.001; *m*/*z* 323: df = 7.035, *t* = 114.821, *P* < 0.001 *t*-test). Compared to the control, labeled glucose was also consumed faster by the fungus in the presence of ammonium (Fig. 4). Even with desugared phloem medium only supplied with d-pinitol (1 g/L), the production of glucose was detected in *L. procerum*-colonized phloem media in the presence of ammonium (Supplementary Fig. S3a, b). However, when ammonium was absent, no glucose was found in *L. procerum*-colonized phloem media (Supplementary Fig. S3a, b). Furthermore, in the *L. procerum*-colonized minimal medium (glucose-free medium with only 1 g/L d-pinitol added), no glucose was produced in either the presence or absence of ammonium (Supplementary Fig. S3c).

### Carbohydrate analysis

The preceding results indicated that ammonium exposure caused the conversion of phloem substances to glucose in *L. procerum*. Thus, we subsequently analyzed the content of glucogenesis-related substances in the phloem. It is well known that pine phloem is rich in cellulose [38]. Other carbohydrates related to glucogenesis in *Pinus tabuliformis* phloem were extracted and analyzed. Starch and sucrose were the only carbohydrates related to glucogenesis detected in the phloem (Table 1). All sample preparations from the phloem (311.87 ± 17.08 mg/g, DW), the phloem medium (236.72 ± 7.03 mg/g, DW) and the desugared phloem (258.61 ± 4.46 mg/g, DW) contained relatively high percentages of starch. Very small amounts of sucrose were present in the sample preparations from the phloem (1.65 ± 0.06 mg/g, DW) and the phloem medium (2.20 ± 0.13 mg/g, DW), and it was not detected in the preparations from the desugared phloem.

**Table 1:** Carbohydrates related to glucogenesis content in *Pinus tabuliformis* phloem, phloem medium, and desugared phloem.

Carbon source	Carbohydrates content (mg/g, DW)		
	Phloem	Phloem medium	Desugared phloem
d-Glucose	10.88 ± 0.62	9.57 ± 0.74	13.38 ± 0.04
Sucrose	1.65 ± 0.06	2.20 ± 0.13	0.00 ± 0.00
Maltose	0.00 ± 0.00	0.00 ± 0.00	0.00 ± 0.00
Trehalose	0.00 ± 0.00	0.00 ± 0.00	0.00 ± 0.00
Starch	311.87 ± 17.08	236.72 ± 7.03	258.61 ± 4.46

**Fig. 5:**
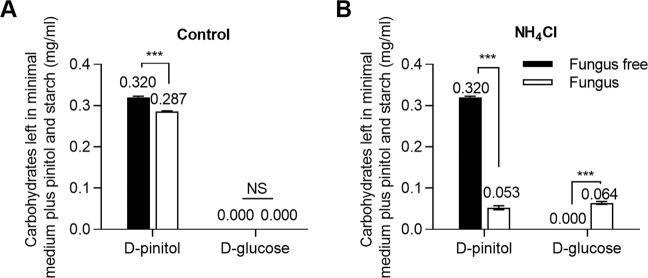
d-Glucose left in the medium is converted from starch. Carbohydrate composition in *L. procerum*-colonized area of minimal media with starch and d-pinitol at day 5 without (**a**) and with (**b**) ammonium. Error bars represent SEs of eight biological replicates. Asterisk indicates significant difference between fungus and fungus-free (*P < 0.05, **P < 0.01, ***P < 0.001). NS means no significance.

No glucose was produced in the *L. procerum*-colonized minimal medium (adding 1 g/L d-pinitol) despite the presence or absence of ammonium (Supplementary Fig. S3c). However, in the minimal medium supplemented with 1 g/L d-pinitol and 20 g/L starch, glucose was detected only in the *L. procerum*-colonized minimal medium with the addition of starch in the presence of ammonium (Fig. 5a, b) (d-glucose: df = 6, *t* = −18.630, *P* < 0.001 *t*-test). In addition, after introducing two other glucogenesis-related carbohydrates (cellulose and sucrose) to the minimal medium (adding 1 g/L d-pinitol), there was still no glucose detected in the minimal medium in the presence of ammonium (Supplementary Fig. S4). Further investigation revealed that *L. procerum* and RTB larvae performed better on minimal media with glucose than that on minimal media with only starch (Supplementary Fig. S5) (df = 42, *t* = 2.957, *P* = 0.008 *t*-test). All of these results indicated that starch is the only substance in phloem available for the production of glucose, and *L. procerum* is able to convert the nutrient-poor but abundant carbohydrate (starch) into a highly nutritional carbon source (glucose).

### The bacterial volatile ammonia, as a nitrogen source, induces the *L. procerum* starch metabolism pathway to convert starch to high nutritional-value glucose

**Fig. 6:**
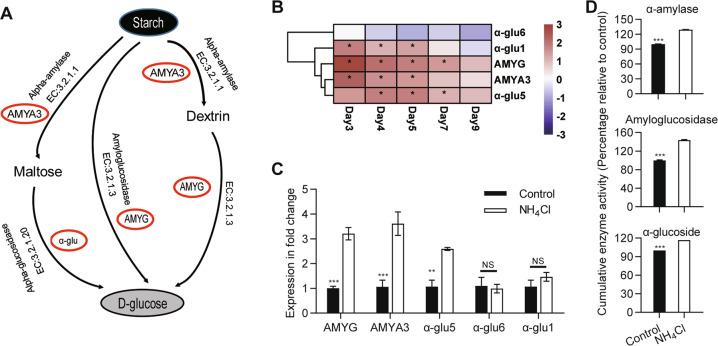
Impacts of ammonium exposure on the gene expression and enzyme activities that related to hydrolyze starch to glucose/the digestion of starch. (**a**) Schematic showing key components of the conversion of starch to glucose. (**b**) Heatmap of genes for hydrolyzing starch to glucose/the digestion of starch at different time point. Asterisk (*) indicated that an absolute value of log_2_Ratio ≥ 1 and FDR < 0.05. (**c**) qRT-PCR validation. All tested samples were collected at day 5. (**d**) Enzymatic activities of amylolytic enzymes. Asterisk of (**c**) and (**d**) indicates significant difference between control and NH_4_Cl treatment (*P < 0.05, **P < 0.01, ***P < 0.001). NS means no significance. Error bars of (**c**) and (**d**) represent SEs of three biological replicates.

As evidenced that starch is responsible for the production of glucose by *L. procerum*, we then used next generation sequencing to determine which gene was involved in this process (Fig. 6a). Genes for hydrolyzing starch to glucose/digestion of starch, including α-amylase (AMYA3), which is involved in the breakdown of long chain carbohydrates, amyloglucanase (AMYG) and α-glucosidase (α-glu1 and α-glu5), which are involved in breaking down starch to the monosaccharide glucose, were upregulated by the exposure of ammonium (Fig. 6b). Other genes related to cellulose and sucrose metabolism showed no response to ammonium exposure (Supplementary Fig. S6). qRT-PCR analyses also found that exposure to ammonium significantly induced the expression of AMYA3, AMYG and α-glu5 (Fig. 6c) (AMYG: df = 4, *t* = −8.402, *P* < 0.001; AMYA3: df = 4, *t* = −4.693, *P* < 0.001; α-glu5: df = 4, *t* = −5.618, *P* = 0.005; α-glu6: df = 4, *t* = 0.288, *P* = 0.787; α-glu1: df = 4, *t* = −1.247, *P* = 0.280 *t*-test).

Besides the expression of these genes, we also measured the enzymatic activity of α-amylase, amyloglucanase, and α-glucosidase by double antibody sandwich methods. Compared to the control, ammonium-treated *L. procerum* showed higher enzymatic activities of α-amylase (df = 4, *t* = −45.369, *P* < 0.001 *t*-test), amyloglucanase (df = 4, *t* = −30.845, *P* < 0.001 *t*-test) and α-glucosidase (df = 4, *t* = −285.833, *P* < 0.001 *t*-test) (Fig. 6d). Taken together, these outcomes strongly indicated that the bacterial volatile ammonia mediated the conversion of starch to glucose via the activation of starch hydrolyzes-related enzymes.

### Deletion of amylolytic enzyme genes causes a growth defect of *L. procerum* and RTB larvae

**Fig. 7:**
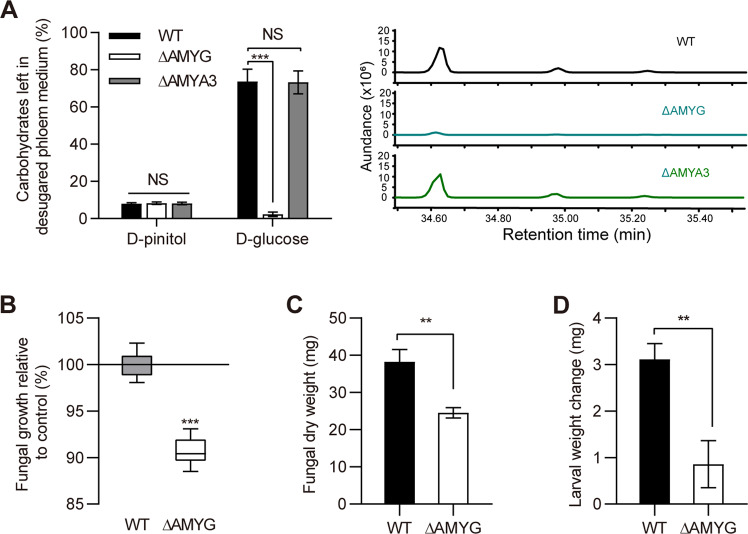
Functional verification of amylolytic enzyme genes for the conversion of starch to glucose. (**a**) Carbohydrate composition in *L. procerum*-colonized area of desugared phloem medium with additional 1 g/L of d-pinitol and d-glucose at day 5 (left) and GC–MS traces of WT, ΔAMYG, and ΔAMYA3 of *L. procerum* on desugared phloem medium (right). Fungal growth (**b**) and dry weight (**c**) of WT and ΔAMYG of *L. procerum* on ammonium infused desugared phloem medium at day 13. (**d**) Effects of exposure of ammonium on the weight change of RTB larvae with the present of WT or ΔAMYG of *L. procerum*. Error bars of (**a**–**c**) represent SEs of eight biological replicates, and error bars of (**d**) represent SEs of at least 20 biological replicates. Asterisk indicates significant difference between WT, ΔAMYG, and ΔAMYA3 of *L. procerum* (*P < 0.05, **P < 0.01, ***P < 0.001). NS means no significance.

Given these findings, we subsequently generated ΔAMYG and ΔAMYA3 mutants of *L. procerum* by homologous recombination. Carbohydrate composition in the desugared phloem medium (adding 1 g/L, d-glucose and d-pinitol) showed that, when exposed to ammonium, the glucose left in the culture medium colonized by ΔAMYG mutant was significantly reduced compared to wild-type (WT), while deletion of AMYA3 was not (Fig. 7a) (AMYG: df = 7.426, *t* = 10.572, *P* < 0.001; AMYA3: df = 14, *t* = 4.248, *P* = 0.180 *t*-test). We found that loss of AMYG led to growth defects of *L. procerum* (Fig. 7b, c) (b: df = 14, *t* = 12.648, *P* < 0.001; c: df = 14, *t* = 4.075, *P* = 0.003 *t*-test), and weight loss of RTB larvae (Fig. 7d) (df = 43, *t* = 3.681, *P* = 0.001 *t*-test). The morphology of ΔAMYG mutant is also different from WT (Supplementary Fig. S7).

### The inhibition of glucose sensing/metabolism and starch metabolism pathway caused by ΔSUC1 mutant

**Fig. 8:**
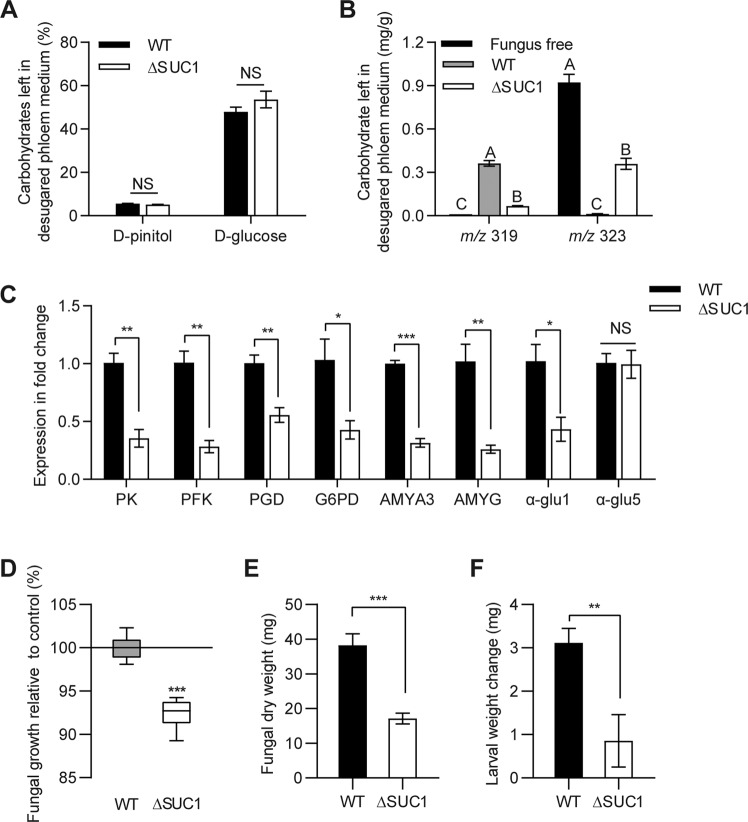
Functional verification of SUC1 for the regulation of starch metabolism. (**a**) Carbohydrate composition in *L. procerum*-colonized area of desugared phloem medium with additional 1 g/L of d-glucose and d-pinitol at day 5. (**b**) Carbohydrate composition in *L. procerum-*colonized area of desugared phloem medium with additional 1 g/L of pinitol and ^13^C_6_-labeled glucose at day 5. (**c**) Expression of genes that involved in glucose and starch metabolism in WT and ΔSUC1 of *L. procerum* on desugared phloem medium at day 5. Fungal growth (**d**) and dry weight (**e**) of WT and ΔSUC1 of *L. procerum* on ammonium infused desugared phloem medium at day 13. (**f**) Effects of exposure of ammonium on the weight change of RTB larvae with the present of WT or ΔSUC1 of *L. procerum*. Error bars of **a**–**c** represent SEs of eight biological replicates, and error bars of (**d**) represent SEs of at least 20 biological replicates. Different letters of (**b**) indicate significant differences among different treatments (*P* < 0.05). Asterisks of **a**, **c**, **d**, **e** and **f** indicate significant difference between WT and ΔSUC1 of *L. procerum* (*P < 0.05, **P < 0.01, ***P < 0.001). NS means no significance.

Having confirmed the conversion of starch to glucose, we then investigated which factors regulate this conversion. A transcription factor deletion of COL-26 and its homology gene has been reported to have a critical role in utilization of starch and integration of carbon and nitrogen metabolism in *Neurospora crassa* and *Trichoderma reesei* [40]. In this work, the homology gene of COL-26 was identified and named as SUC1 in *L. procerum* (Supplementary Fig. S8). When compared to WT strains, ΔSUC1 mutants showed growth defects in the presence of ammonium and desugared phloem medium, which also had an extra 1 g/L of d-glucose-^13^C_6_ and d-pinitol added (Supplementary Fig. S8a, b). To investigate the functions of SUC1 in this process, we analyzed the carbohydrate composition in the plates and evaluated transcriptional changes in the ΔSUC1 mutant when switched to desugared phloem medium containing 1 g/L of d-glucose-^13^C_6_ and d-pinitol under identical conditions as with the WT parent strain (see above). We found that loss of SUC1 has no effect on the content of pinitol and glucose in desugared phloem medium (adding 1 g/L of d-glucose-^13^C_6_ and d-pinitol) following fungus consumption in the presence of ammonium (Fig. 8a) (d-pinitol: df = 14, *t* = 1.87, *P* = 0.086; d-glucose: df = 14, *t* = −1.277, *P* = 0.226 *t*-test). Furthermore, glucose left in plates with ΔSUC1 mutant was mainly labeled glucose. In contrast, glucose left in plates with WT mainly was natural glucose (Fig. 8b and Supplementary S9) (*m*/*z* 319: *F*_2, 21_ = 228.376, *P* < 0.001; *m*/*z* 323: *F*_2, 21_ = 162.146, *P* < 0.001 ANOVA, Tukey’s test). Based on the transcription analysis, we found that the main genes in the glucose regulon gene set and starch regulon gene set were downregulated in the ΔSUC1 mutant (Fig. 8c) (PK: df = 4, *t* = 5.745, *P* = 0.005; PFK: df = 4, *t* = 6.450, *P* = 0.003; PGD: df = 4, *t* = 4.752, *P* = 0.009; G6PD: df = 4, *t* = 3.076, *P* = 0.037; AMYA3: df = 4, *t* = 14.602, *P* < 0.001; AMYG: df = 4, *t* = 4.983, *P* = 0.008; α-glu1: df = 4, *t* = 3.315, *P* = 0.030; α-glu5: df = 4, *t* = 0.085, *P* = 0.936 *t*-test), including two key genes of the glycolysis (6-phosphogluconate dehydrogenase: PGD and glucose 6‐phosphate dehydrogenase: G6PD), two key genes of the pentose phosphate pathway (pyruvate kinase: PK and phosphofructokinase: PFK), and three amylolytic genes (AMYA3, AMYG and α-glu1). In addition, deletion of SUC1 also impaired the growth of *L. procerum* (Fig. 8d, e) (d: df = 14, *t* = 10.25, *P* < 0.001; e: df = 14, *t* = 5.785, *P* < 0.001 *t*-test) and RTB larvae (Fig. 8f) (df = 33.878, *t* = 3.276, *P* = 0.002 *t*-test) significantly. The morphology of ΔSUC1 mutant was different from WT as well (Supplementary Fig. S7). These data indicated that deletion of the SUC1 repressed the glucose sensing/metabolism and starch metabolism pathway in *L. procerum* with ammonium, which therefore disrupted the beneficial association of *L. procerum* and RTB larvae.

## Discussion

Many aggressive beetles infesting conifers over a wide geographical range depend upon associations with fungi to fulfill a range of physiological and ecological functions [52–54]. Our previous studies have shown that *D. valens–L. procerum* formed a mutualistic beetle–fungus invasive complex under the nutrient-poor bark, and this mutualism is consistently maintained for multiple generations [55]. However, competition for nutritional resources is ultimately unavoidable. The symbiotic bacteria volatile ammonia regulates carbohydrate consumption in *L. procerum* to eliminate the negative effects on the RTB larval growth caused by the carbohydrate competition of *L. procerum*, yet little was known prior to this study about the mechanism behind this phenomenon. Based on our results, we propose that *D. valens* maintains mutualistic associations with the beneficial symbiont *L. procerum* under poor nutrition with the symbiotic bacterial volatile regulating the mutualistic fungus’ carbohydrate metabolism. On one hand, the carbohydrate consumption of *L. procerum* is accelerated (Fig. 4), which is beneficial for its competition with antagonistic fungi while the carbohydrate consumption of antagonistic fungi was significantly reduced by symbiotic bacteria [17]. Moreover, it induces the *L. procerum* starch metabolism pathway to secret extracellular amylase (Fig. 6), converting the abundant starch in the phloem into a high nutritional-value carbon source (glucose) (Fig. 5 and Supplementary Fig. S5), and providing sufficient preferred carbon source for the symbionts, maintaining the stability of the *D. valens*–*L. procerum* mutualism [47]. As glucose is a better carbon source for these associated bacteria, the conversion not only replenishes the energy cost of releasing ammonia but also provides more energy for its growth [47]. These results show carbohydrate metabolism mediated by associated bacteria–fungi interactions can have a key role in maintaining the bark beetle–fungus mutualism in face of nutritional limitation. In addition, we find that the transcription factor, SUC1, is an essential regulator for the process (Fig. 8). Our RNA-sequence data also showed that ammonia nitrogen is converted into glutamate by glutamate dehydrogenase (Supplementary Fig. S10), and further investigation is needed to explore the full extent of how ammonia is used as a nitrogen source [37]. Notably, the homolog of SUC1 in *N. crassa*, COL-26, was reported to have a role in coordinating the utilization of starch components with nitrogen regulation [40].

In nature, ten fungal species were found in association with RTB, while only *L. procerum* was consistently found with *D. valens* at all collection sites and the isolation frequency of this species was the highest in China [42, 43]. Due to the consistent relationship and potential to facilitate the invasion of RTB, *L. procerum* has been proven to be a symbiotic fungus for RTB [17]. In pine phloem, carbon sources available for both fungal and bacterial symbionts are mainly d-pinitol followed by d-glucose [31]. Previous studies have shown that dominant bacteria associated with RTB and their released volatiles, including ammonia, inhibit the growth and main carbohydrates consumption of antagonistic fungi *O. minus*, which alleviate antagonistic effects of *O. minus* on RTB larvae [37, 47, 56]. On the contrary, in this study, the volatile ammonia from bacteria associated with RTB accelerates the consumption of the main carbohydrates by mutualistic fungi *L. procerum*. The growth of *L. procerum* is significantly enhanced by the acceleration of carbohydrate consumption. This enhancement might have resulted in higher pathogenicity to Chinese host pines since *L. procerum* is generally considered as a weakly pathogenic fungus, thereby facilitating the invasion of RTB by helping to overcome tree defenses [17]. Taken together, all of these results indicated that accelerated consumption of carbohydrates by the mutualistic fungi *L. procerum* improves the chances of this mutualistic fungal symbiont to outcompete other microbial competitors and leads *L. procerum* to be the dominant fungus in the gallery with different life stages of RTB [42]. The enhancement of growth and competitiveness of *L. procerum* is potentially conducive to the successful invasion of RTB.

The mutualistic fungi *L. procerum* has been revealed to be beneficial to the colonization and invasion of the RTB [17]. However, the competition for carbon source via carbohydrate competition could drastically inhibit RTB larval growth and then indirectly affect bark beetle population dynamics [31]. The accelerated consumption of glucose by fungi *L. procerum* would inevitably affect the maintenance of the *D. valens*–*L. procerum* mutualism. However, RTB successfully maintains beneficial fungi under natural conditions in the field, which suggests that the mutualism has a strategy to overcome the disadvantages of nutritional competition. Our current study demonstrates that the associated bacteria can alleviate the antagonistic effects of the fungus *L. procerum* on RTB larval growth with the volatile ammonia regulating carbon source usage in the fungus. Tripartite beetle–fungus–bacterium mutualisms are widespread in nature, e.g., *Dendroctonus frontalis* uses associated bacteria to protect its fungal food source from a competitor fungus to maintain the *D. frontalis*–*Entomocorticium* sp. mutualism [2]. In our study, we found that the bacterial volatile ammonia, as a nitrogen source, triggered *L. procerum* to convert the phloem’s abundant starch resources, normally not easily accessible to RTB, into glucose by the activation of starch metabolism pathways, while elimination of the conversion by AMYG deletion impaired the growth of *L. procerum* and RTB larvae. Thus, we suggest that the conversion of starch into glucose compensates the acceleration of carbohydrate consumption, and subsequently alleviates the nutrient competition between beneficial fungi and bark beetles.

The preceding results showed that the volatile ammonia not only accelerated the consumption of glucose but also triggered conversion of starch into glucose in *L. procerum*. The acceleration of carbohydrate consumption and the conversion of starch into glucose benefit this invasive beetle–fungus complex by alleviating nutrition-limitation in the presence of RTB-associated bacteria. However, the molecular mechanism of how the volatile ammonia influences the metabolic ability of *L. procerum* remains unknown. In *N. crassa*, the transcription factor COL-26 was reported to have a role in coordinating the utilization of starch components with nitrogen regulation [40]. Here, we found a homolog of COL-26 in *L. procerum*, SUC1. The ΔSUC1 mutant showed a defect on converting starch to glucose and inhibited the utilization of glucose. All of these results demonstrated that the volatile ammonia influenced the metabolism of *L. procerum* by regulating the expression of SUC1, which functions as a mediator of glucose signaling and metabolism.

Overall, we defined a novel nutrient consumption–compensation strategy to illustrate how insects can maintain mutualistic associations with beneficial symbionts under nutritional stress. Our results revealed that the nutrient consumption–compensation strategy, mediated by tripartite beetle–fungus–bacterium interactions, benefits the invasive bark beetle by facilitating the maintenance of its mutualistic associations with beneficial fungus under poor nutritional conditions. As nutritional competition is inevitable in the case of a nutrient-poor environment, this nutrient consumption–compensation strategy may prove to be ubiquitous among many species of bark beetles and their symbiotic fungi [9]. Furthermore, several investigations have revealed that the phloem of bark is nitrogen-limited and bark beetle-associated microbes could help ease nitrogen limitation [9, 22, 57]. In this work, we found that bacterial ammonia serves as a nitrogen source to regulate the stability of the bark beetle–fungus complex, suggesting that nitrogen fertilizers used in farming might affect the stability of other insect–microbe symbionts in plantation forests or agriculture. Understanding the consumption–compensation strategy not only provides novel insights into the maintenance of insect–microbes mutualisms, but also may lead to the exploration of new management strategies for these devastating forest pests.

## Supplementary information

Supplementary information
