# Supplementary material for: An invasive beetle–fungus complex is maintained by fungal nutritional-compensation mediated by bacterial volatiles
Source: ISME J. 2020 Aug 19;14(11):2829–42. doi: 10.1038/s41396-020-00740-w (PMC7784882; doi:10.1038/s41396-020-00740-w)
Supplement: Supplementary file 1 — Supplementary information [file 41396_2020_740_MOESM1_ESM.docx]

**An invasive beetle–fungus complex is maintained by fungal nutritional-compensation mediated by bacterial volatiles**

Fanghua Liu, Jacob D Wickham, Qingjie Cao, Min Lu, Jianghua Sun

**Supplementary Information**

**Contents**

[Supplementary Methods 2](#_Toc45293948)

[Table S1. Primers used in this study 6](#_Toc45293949)

[Fig. S1. Carbohydrate composition in the whole plates of desugared phloem medium without (a) and with (b) ammonium. 8](#_Toc45293950)

[Fig. S2. GC-MS traces of natural glucose (a) and ^13^C_6_-labeled glucose (b). 9](#_Toc45293951)

[Fig. S3. Verification of the synthesis of glucose on restricted medium. 10](#_Toc45293952)

[Fig. S4. Carbohydrate composition in *L. procerum* colonized area of minimal medium with ammonium and cellulose (a) or sucrose (b) at 5th days. 11](#_Toc45293953)

[Fig. S5. Effects of D-glucose and starch on the growth of *L. procerum*, and RTB larvae. 12](#_Toc45293954)

[Fig. S6. Heatmap of gluconeogenesis related genes for cellulose and sucrose. 13](#_Toc45293955)

[Fig. S7. Representative growth of WT, ΔAMYG mutant and ΔSUC1 mutant of *L. procerum* on phloem medium infused with ammonium (0.019 mol/L of NH_4_Cl). 14](#_Toc45293956)

[Fig. S8. Prediction of SUC1 in *L. procerum*. 15](#_Toc45293957)

[Fig. S9. Functional verification of SUC1. 16](#_Toc45293958)

[Fig. S10. Impacts of ammonium exposure on the gene expression related to utilization of ammonia in nitrogen metabolism. 17](#_Toc45293959)

**Supplementary Methods**

**Ammonia, ammonium and Fungal Growth in the Phloem Medium**

Based on these previous results, we selected 1.56 mol/L ammonia and 0.019 mol/L ammonium to assess the effect of the presence or absence of ammonia/ammonium within the phloem medium on the growth performance of *L. procerum.* For technical reasons, we could not measure the concentrations of ammonia present in the RTB gallery, because once the bark is penetrated to locate RTB, ammonia releases to the air immediately. Therefore, based on these previous results, we selected 1.56 mol/L ammonia and 0.019 mol/L ammonium to assess the effect of the presence or absence of ammonia/ammonium within the phloem medium on the growth performance of *L. procerum*. The phloem medium was prepared by mixing 20 g of dry *P. tabuliformis* phloem powder and 10 g of agar followed by 300 mL of distilled water, which was autoclaved (30 min, 120 ^o^C, 0.14 Mpa) then poured into petri dishes [1]. For ammonia test, we used 90 mm Petri dishes that were divided into two equal parts down the middle. *L. procerum* was inoculated on one side of the Petri dish, and 200 μl of 1.56 mol/L ammonia water inside a vial insert was put into the side opposite of the fungi in these Petri dishes. Sterile water was used in the control group. One plug of agar (3 mm diameter) from the leading edge of fungi culture growing on MEA was inoculated onto the phloem medium plate. The plug was placed about 1 cm from the division of the dish, and about 1 cm away from the exterior edge of the dish. Fungal linear growth along the plastic division was measured from the point of inoculation to the leading edge of the hyphae every two days, and all treatments were replicated 20 times. At 13 d post-inoculation, the mycelial mat was gently scraped from the agar by disposable blades, and its fresh weight was determined. The mycelia were then allowed to dry at 50 °C until the weight was constant. The density of the mycelia, determined by the degree of overlap of the hyphae, was measured by spectroscope (Keyence VHX-1000C / Nikon Ni-E / AvaSpec-ULS2048-USB2). Pictures were randomly taken from the area of the mycelial mat at a distance of 1 cm. Using the same approach with minor modifications, the effects of ammonium were examined on *L. procerum* by adding ammonium chloride (99.5%; Pharmco-AAPER) to the phloem medium to achieve concentrations of 0.019 mol/L. Sterile distilled water without ammonium chloride was used for the control. A mycelial plug (3 mm in diameter) of *L. procerum* growing on MEA was transferred to the center of each plate. All of the cultures were grown under 25 ^o^C and 70% RH in darkness for 13 days until the mycelia covered all of the medium. For fungal weight and density assays, at least eight biological replicates were conducted.

**Ammonia, ammonium and *D. valens* larval growth in the phloem medium**

The third instar RTB larvae were surface sterilized with ethanol and transferred into phloem medium for 7 days. Active larvae were selected and pre-starved for 30 min, and then one larva was transferred to each Petri dish. First, the fungal strain was inoculated on phloem medium and incubated at 25 ^o^C and 70% RH in darkness for 10 days until the mycelia covered most of the medium. Surface-sterilized RTB larvae were fed medium with and without *L. procerum* (treatment and control). Second, the fungal strain *L. procerum* was inoculated on phloem medium in the presence or absence of ammonia or ammonium. Fungus-colonized medium was regarded as control medium, while the phloem medium which was colonized by *L. procerum* in the presence of ammonia or ammonium was regarded as treatment. After the mycelia nearly covered the whole medium, these fungus-colonized phloem media were used to feed RTB larvae to test their effects on larval growth as described above. For the starch vs. glucose comparison, n = 32 larvae were used per treatment; all other treatments used n = 25 larvae. Petri dishes were incubated at 25 °C for 6 days, after which specimens within each dish were quantified.

**Preparation of desugared phloem medium**

The desugared media was obtained by methanol extraction of the main carbohydrates in standard phloem media [2]. Specifically, 50g of phloem powder was put into 200 mL of methanol and shaken at 70 ^o^C for 30 min. Then it was filtered by 5 layers of gauze before cooling. After cooling, the phloem powder was resuspended in distilled water, and then filtered by 5 layers of gauze. The phloem powder was treated three times as described above and dried before further experiments. After the elimination, carbohydrate composition in desugared phloem powder was tested before experiments to make sure that no D-pinitol or D-glucose was left [2]. Pure D-glucose 1g/L and D-pinitol 1g/L were added back to the desugared media according to their total content in standard phloem media [2]. Particularly, the desugared phloem medium with ammonium added was set as treatment and medium without ammonium was set as control. The whole desugared phloem medium, and the desugared phloem medium with fungus were sampled at 0, 2, 3, 4, 5, 7, 9, 11, 13 d after the fungus was inoculated. The *L. procerum*-colonized area of phloem media was cut into a small piece (1×1 cm) using sterile disposable blades, and then the entire sample was ground into powder. Carbohydrate composition analyses used a 200 mg sample from each treatment as previously described [2]. We collected one sample per plate and used eight plates per time point (8 biological replicates). In addition, the mycelial mat was scraped for RNA-seq at 3, 4, 5, 7 and 9 days after the exposure of ammonium, which was replicated 3 times.

**RNA-seq Analyses**

The mycelial mat was scraped and flash frozen in liquid nitrogen at 3, 4, 5, 7 and 9 days after the fungus was inoculated. RNA was extracted using the Trizol method (Invitrogen). RNA degradation and contamination was monitored on 1% agarose gels. RNA purity was checked using the NanoPhotometer® spectrophotometer (IMPLEN, CA, USA). RNA concentration was measured using Qubit® RNA Assay Kit in Qubit® 2.0 Flurometer (Life Technologies, CA, USA). RNA integrity was assessed using the RNA Nano 6000 Assay Kit of the Bioanalyzer 2100 system (Agilent Technologies, CA, USA). Three biological repeats were established for each treatment.

A total amount of 1 µg RNA per sample was used as input material for the RNA sample preparations. Sequencing libraries were generated using NEBNext® Ultra™ RNA Library Prep Kit for Illumina® (NEB, USA) following manufacturer’s recommendations and index codes were added to attribute sequences to each sample. Briefly, mRNA was purified from total RNA using poly -T oligo-attached magnetic beads. Fragmentation was carried out using divalent cations under elevated temperature in NEB Next First Strand Synthesis Reaction Buffer (5X). First strand cDNA was synthesized using random hexamer primer and M-MuLV Reverse Transcriptase (RNase H-). Second strand cDNA synthesis was subsequently performed using DNA Polymerase I and RNase H. Remaining overhangs were converted into blunt ends via exonuclease/polymerase activities. After adenylation of 3’ ends of DNA fragments, NEBNext Adaptor with hairpin loop structure were ligated to prepare for hybridization. In order to select cDNA fragments of preferentially 250~300 bp in length, the library fragments were purified with AMPure XP system (Beckman Coulter, Beverly, USA). Then 3 µl USER Enzyme (NEB, USA) was used with size-selected, adaptor-ligated cDNA at 37°C for 15 min followed by 5 min at 95 °C before PCR. Then PCR was performed with Phusion High-Fidelity DNA polymerase, Universal PCR primers and Index (X) Primer. At last, PCR products were purified (AMPure XP system) and library quality was assessed on the Agilent Bioanalyzer 2100 system.

The clustering of the index-coded samples was performed on a cBot Cluster Generation System using TruSeq PE Cluster Kit v3-cBot-HS (Illumia) according to the manufacturer’s instructions. After cluster generation, the library preparations were sequenced on an Illumina Hiseq platform and 125 bp/150 bp paired-end reads were generated.

Raw data (raw reads) of fastq format were firstly processed through in-house perl scripts. In this step, clean data (clean reads) were obtained by removing reads containing adapter, reads containing ploy-N and low quality reads from raw data. At the same time, Q20, Q30 and GC content the clean data were calculated. All the downstream analyses were based on the clean data with high quality. Reference genome and gene model annotation files were provided by Dr Min Lu (Unpublished). Index of the reference genome was built using Hisat2 v2.0.4 and paired end clean reads were aligned to the reference genome using Hisat2 v2.0.4 [3]. We selected Hisat2 as the mapping tool for that Hisat2 can generate a database of splice junctions based on the gene model annotation file and thus a better mapping result than other non-splice mapping tools. HTSeq v0.9.1 was used to count the reads numbers mapped to each gene [4, 5]. And then FPKM of each gene was calculated based on the length of the gene and reads count mapped to this gene. FPKM, expected number of Fragments Per Kilobase of transcript sequence per Millions base pairs sequenced, considers the effect of sequencing depth and gene length for the reads count at the same time, and is currently the most commonly used method for estimating gene expression levels [6]. Differential expression analysis of two conditions/groups (two biological replicates per condition) was performed using the DESeq R package (1.18.0) [7, 8]. DESeq provide statistical routines for determining differential expression in digital gene expression data using a model based on the negative binomial distribution. The resulting P-values were adjusted using the Benjamini and Hochberg’s approach for controlling the false discovery rate. Genes with an adjusted P-value <0.05 found by DESeq were assigned as differentially expressed. Gene Ontology (GO) enrichment analysis of differentially expressed genes was implemented by the GOseq R package, in which gene length bias was corrected [9]. GO terms with corrected P-values less than 0.05 were considered significantly enriched by differential expressed genes. KEGG is a database resource for understanding high-level functions and utilities of the biological system, such as the cell, the organism and the ecosystem, from molecular-level information, especially large-scale molecular datasets generated by genome sequencing and other high-through put experimental technologies (http://www.genome.jp/kegg/) [10]. We used KOBAS software to test the statistical enrichment of differential expression genes in KEGG pathways [11]. Transcriptome data are available in NCBI bioproject PRJNA637686.

**RNA preparation and qRT-PCR analysis**

Total RNA of fungus was extracted by Trizol reagent (Life Technologies, USA) following the standard protocol. For quantitative RT-PCR, complementary DNAs (cDNAs) were synthesized with total RNAs (800 ng per reaction) using Fast Quant RT Kit (Tiangen, China). Ten times diluted cDNAs were used as templates for qRT-PCR analysis with SYBR® Premix Ex Taq™ without ROX (TaKaRa, Japan). The genes *tubulin* was used as internal standards. The relative expression level of each gene was determined using the 2^−ΔΔCt^ method. All qRT-PCR were repeated three times.

**Gene knockout in *L. procerum***

Briefly, the 5' and 3' flanking sequences of SUC1, AMYG and AMYA3 genes were amplified using different primer pairs (Table S1) with the PhantaTM Super-Fidelity DNA Polymerase (Vazyme, Piscataway, NJ). PCR products were digested with respective restriction enzymes (Table S1) and then inserted into the corresponding sites of the binary vector pDHt-Hyg to generate the disruption plasmids for Agrobacterium-mediated transformation (AMT) of *L. procerum*. The hph gene from vector pDHt-hyg was used for selection maker after transformation. PCR and RT-PCR were performed to verify the success of gene deletion. Reference gene, *β*-Tubulin, was also amplified as an internal positive control. The influence of gene deletion on the metabolism of carbohydrate of *L. procerumm*, and the performance of *L. procerumm* and RTB larvae were conducted as previously described.

**Reference**

1. Zhou FY, Xu LT, Wang SS, Wang B, Lou QZ, Lu M, et al. Bacterial volatile ammonia regulates the consumption sequence of D-pinitol and D-glucose in a fungus associated with an invasive bark beetle. ISME J. 2017;11(12):2809-2820.
2. Zhou FY, Lou QZ, Wang B, Xu LT, Cheng CC, Lu M, et al. Altered carbohydrates allocation by associated bacteria-fungi interactions in a bark beetle-microbe symbiosis. Sci Rep. 2016;6:20135.

3. Kim D, Langmead B, Salzberg SL. HISAT: a fast spliced aligner with low memory requirements. Nat Methods. 2015;12:357.

4. Anders S, Pyl PT, Huber W. HTSeq: Analysing high-throughput sequencing data with Python. 2010.

5. Anders S, Pyl PT, Huber W. HTSeq-a Python framework to work with high-throughput sequencing data. Bioinformatics. 2015;31:166-169.

6. Trapnell C, Williams BA, Pertea G, Mortazavi A, Kwan G, van Baren MJ, et al. Transcript assembly and quantification by RNA-Seq reveals unannotated transcripts and isoform switching during cell differentiation. Nat Biotechnol. 2010;28:511.

7. Anders S, Huber W. Differential expression analysis for sequence count data. Genome Boil**.** 2010;11:R106.

8. Anders S, Huber W. Differential expression of RNA-Seq data at the gene level–the DESeq package. Available at www.bioconductor.org/packages/devel/bioc/vignettes/DESeq/inst/doc/DESeq.pdf. 2012;Accessed October 17, 2013.

9. Young MD, Wakefield MJ, Smyth GK, Oshlack A. Gene ontology analysis for RNA-seq: accountting for selection bias. Genome Biol. 2010;11:R14.

10. Kanehisa M, Araki M, Goto S, Hattori M, Hirakawa M, Itoh M. KEGG for linking genomes to life and the environment. Nucleic Acids Res. 2007;36(suppl_1):D480-D484.

11. Mao X, Cai T, Olyarchuk JG, Wei L. Automated genome annotation and pathway identification using the KEGG Orthology (KO) as a controlled vocabulary. Bioinformatics. 2005;21:3787-3793 (2005).

# **Table S1. Primers used in this study**

| Gene | Primers | | Primer sequences | Restriction  enzymes | Length  (bp) | | Application |
| --- | --- | --- | --- | --- | --- | --- | --- |
| hph | hph -F | | ATGAAAAAGCCTGAACTCACCG |  | 811 | | Verification |
|  | hph-R | | GACCAATGCGGAGCATATACG |  |  |  |  |
| AMYA3 | Amya-UF | atcgaattcctgcagcccgggTCTAATCGATATGGCCGCTATGA | | SmaI | 2100 | Gene deletion | |
|  | Amya-UR | taattgcgcggatcccccgggGATCCTGTGGACTGTGTGTGTTTATT | | SmaI |  |  |  |
|  | Amya-DF | ccagccctgactagttctagaGCGTGTGTCAACGAGAGCCT | | XbaI | 2100 |  |  |
|  | Amya-DR | cgcggtggcggccgctctagaTCGACGAGATCAGCATGATTGA | | XbaI |  |  |  |
|  | ISSR-Aya-UF | CGCTGTTGAGGCTGTAGATG | |  | 3184 | Verification | |
|  | ISSR-Aya-UR | GATGCAATAGGTCAGGCTCTC | |  |  |  |  |
|  | ISSR-Aya-DF | TCGGGCGTACACAAATCG | |  | 3052 |  |  |
|  | ISSR-Aya-DR | ACTCAACGAACCCGACGAA | |  |  |  |  |
|  | Amya3 F | ATGAAGCTGTCTCGCATC | |  | 109 | Verification/ qRT-PCR | |
|  | Amya3 R | ACCGGTCTGTCAGCAAAA | |  |  |  |  |
| AMYAG | Amyg-UF | | atcgaattcctgcagcccgggACGGAGGAGCAGAAGAAGTGG | SmaI | 1972 | | Gene deletion |
|  | Amyg-UR | | taattgcgcggatcccccgggTCGTTAAGCTAAGTAGCGGTATTGG | SmaI |  |  |  |
|  | Amyg-DF | | ccagccctgactagttctagaCTATGTCTCTGGCATCTGTTCCTTT | XbaI | 1997 | |  |
|  | Amyg-DR | | cgcggtggcggccgctctagaGATGTCCTGCACCGCTGTACTG | XbaI |  |  |  |
|  | ISSR-Ayg-UF | | GTCCCGTGCCTGCATACAT |  | 3013 | | Verification |
|  | ISSR-Ayg-UR | | GATTCTTCGCCCTCCGAGAG |  |  |  |  |
|  | ISSR-Ayg-DF | | CCGAGGGCAAAGGAATAGAG |  | 3013 | |  |
|  | ISSR-Ayg-DR | | TCGCCGAAGAAGAGAAAGG |  |  |  |  |
|  | Amyg-F | | GGCGAACCAGAGATTGTTAGAA |  | 105 | | Verification/qRT-PCR |
|  | Amyg-R | | GGCGAACCAGAGATTGTTAGAA |  |  |  |  |
| SUC1 | Suc-UF | | atcgaattcctgcagcccgggGATGTACAGAACGGCGAAGTTTT | SmaI | 1959 | | Gene deletion |
|  | Suc-UR | | taattgcgcggatcccccgggAGCAGTGGATGAATTTCAGATCC | SmaI |  |  |  |
|  | Suc-DF | | ccgtcaccagccctgactagtTGGGAAGCAGGGCGACAA | SpeI | 2019 | |  |
|  | Suc-DR | | ggcggccgctctagaactagtTACGACCACGAAGGCCAAGA | SpeI |  |  |  |
|  | ISSR-Suc-UF | | AACACCTACGTTTAGAGATTCG |  | 3020 | | Verification |
|  | ISSR-Suc-UR | | ATTCTTCGCCCTCCGAGAG |  |  |  |  |
|  | ISSR-Suc-DF | | CGAGGGCAAAGGAATAGAGTAG |  | 3003 | |  |
|  | ISSR-Suc-DR | | GATCTATCGCATCCCATAACGG |  |  |  |  |
|  | SUC1-F | | ATTGTCACCGCCTCCTCTG |  | 115 | | Verification/qRT-PCR |
|  | SUC1-R | | CTGTTGTTGCTGCCGTTGA |  |  |  |  |
| *β*-tubulin | Tub-1F | | GACTTGCTCTGCCATCTT |  | 298 | | RT-PCR reference gene |
|  | Tub-1R | | CTCCGTGAACTCCATCTC |  |  |  |  |
|  | Tub-F | | ACTCGTTCCGTGCTATCA |  | 148 | | qRT-PCR reference gene |
|  | Tub-R | | TCCTCTACCTCCTTCATCG |  |  |  |  |
| AMYA3 | Amya3-F | | ATGAAGCTGTCTCGCATC |  | 109 | | qRT-PCR |
|  | Amya3-R | | ACCGGTCTGTCAGCAAAA |  |  |  |  |
| α-glu5 | Glu5-F | | ATGTCTCCCCACCTAGTCG |  | 123 | | qRT-PCR |
|  | Glu5-R | | TCCCTTCAGGTCTCCAGTG |  |  |  |  |
| α-glu6 | Glu6-F | | CTCAGCAAGGTGGACTAC |  | 104 | | qRT-PCR |
|  | Glu6-R | | TAGTTGGAGATGTCGTAGC |  |  |  |  |
| α-glu1 | Glu1-F | | GTGGTAGCATTAGCTGGC |  | 112 | | qRT-PCR |
|  | Glu1-R | | AAGAAGCTCCGAGGGAGAG |  |  |  |  |
| PK | PK-F | | CTCTGCTCTCGACCACCTCT |  | 104 | | qRT-PCR |
|  | PK-R | | AGATGATGGATGTCCGGC |  |  |  |  |
| PFK | PFK-F | | ATGACAAGCATCACCACAC |  | 100 | | qRT-PCR |
|  | PFK-R | | ATATTCCCGTGCCTCCAG |  |  |  |  |
| G6PD | G6PD-F | | ATGGAGGACTCTGGCATG |  | 102 | | qRT-PCR |
|  | G6PD-R | | AAACAGGGCCGGATACGT |  |  |  |  |
| PGD | PGD-F | | CGTCATGGGCCAAAACCT |  | 102 | | qRT-PCR |
|  | PGD-R | | TCATTGGCCAGGAATCTG |  |  |  |  |


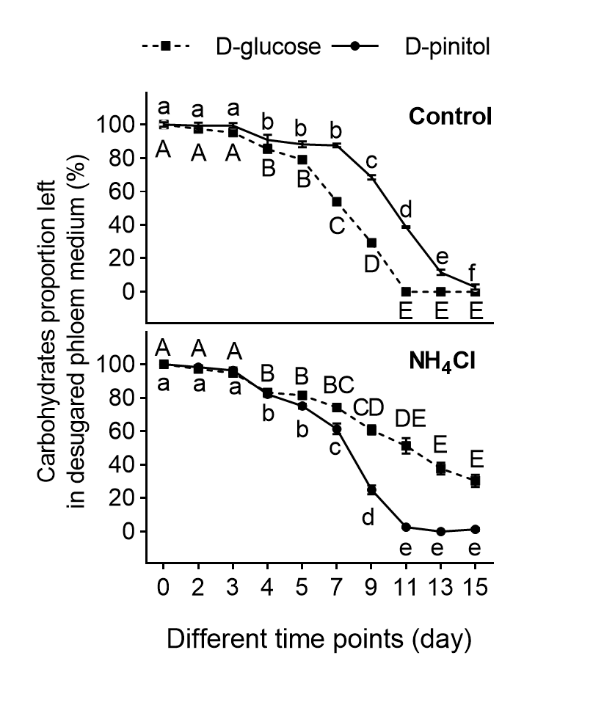


**Fig. S1 Carbohydrate composition in the whole plates of desugared phloem medium without (a) and with (b) ammonium.** Different letters indicate significant differences among different treatments (*P* < 0.05).


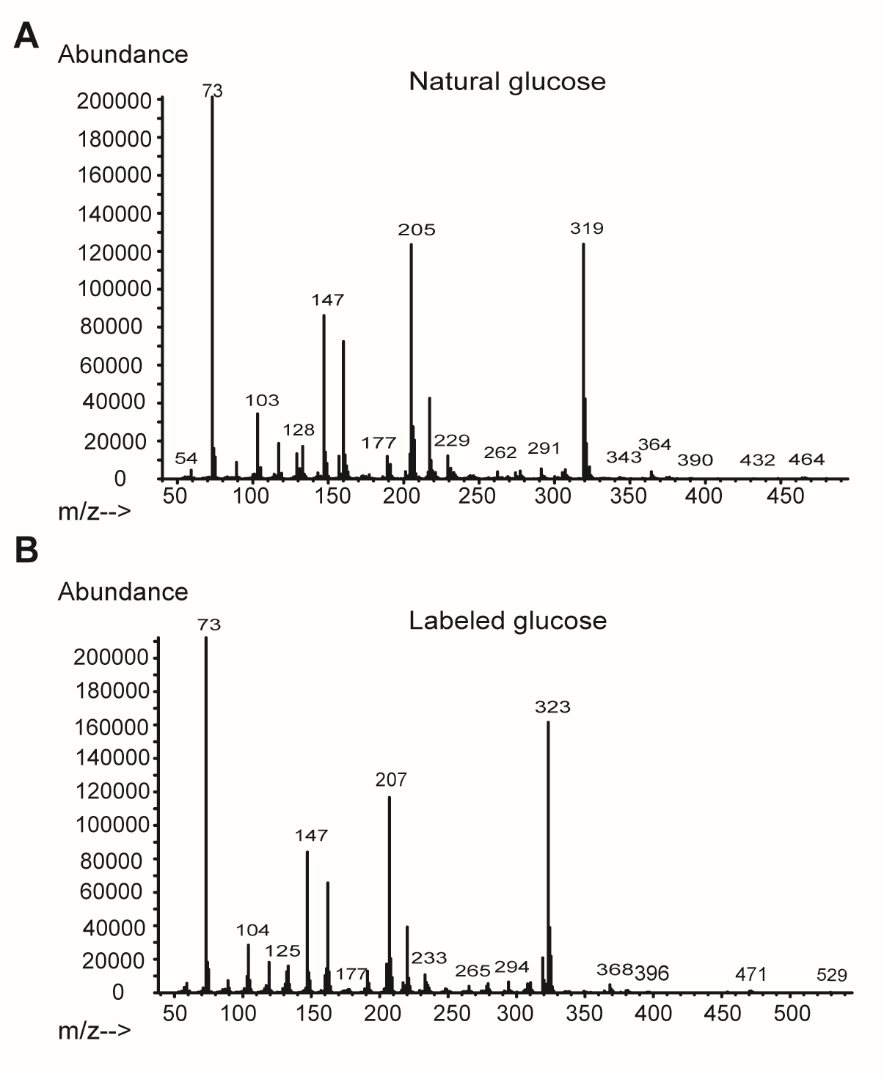


# **Fig. S2 GC-MS traces of natural glucose (a) and ^13^C_6_-labeled glucose (b).**


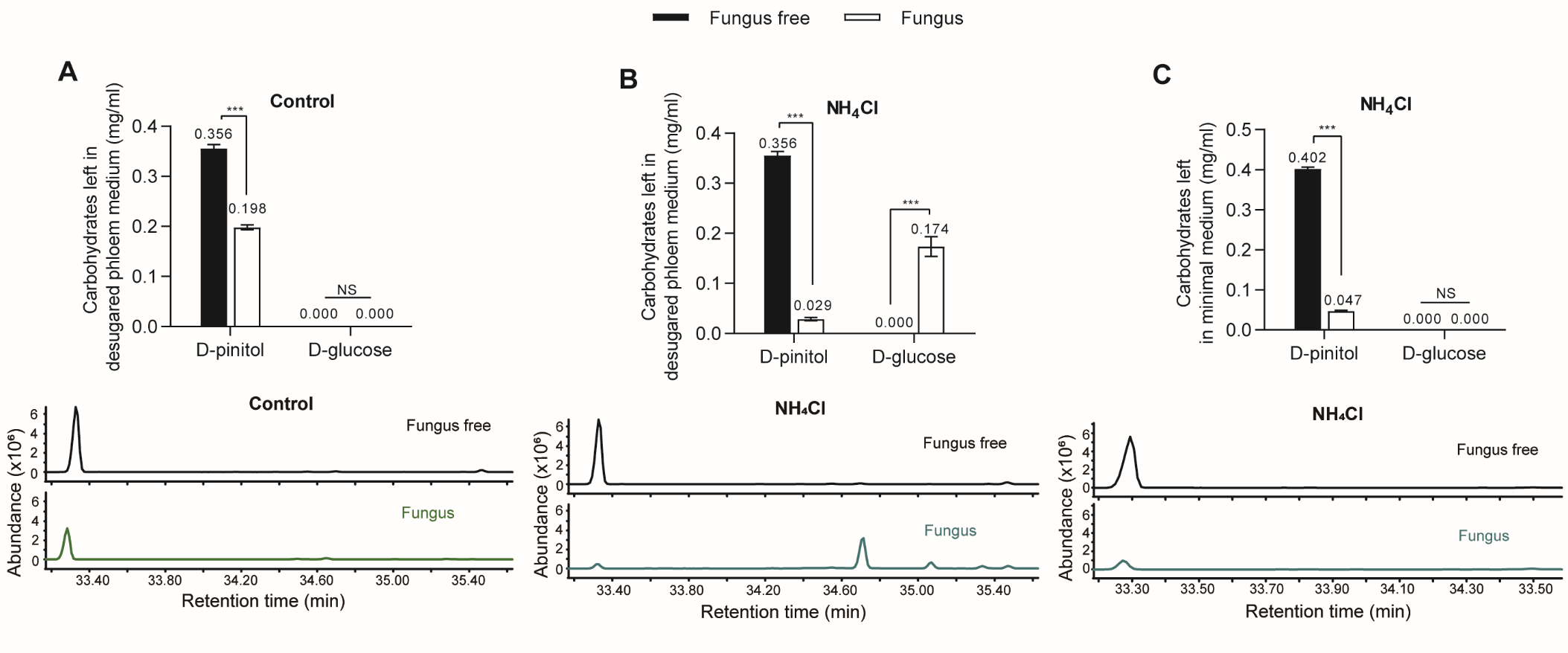


**Fig. S3 Verification of the synthesis of glucose on restricted medium. a** Carbohydrate composition (*Top*) and GC-MS traces (*Bottom*) in *L. procerum* on desugared phloem medium with additional D-pinitol at day 5. **b** Carbohydrate composition (*Top*) and GC-MS traces (*Bottom*) in *L. procerum* on desugared phloem medium with additional D-pinitol and ammonium at day 5. **c** Carbohydrate composition (*Top*) and GC-MS traces (*Bottom*) in *L. procerum* on minimal medium with additional D-pinitol and ammonium at day 5. Asterisk indicates significant difference between fungus and fungus free (* *P* < 0.05, ** *P* < 0.01, *** *P* < 0.001). NS means no significance.


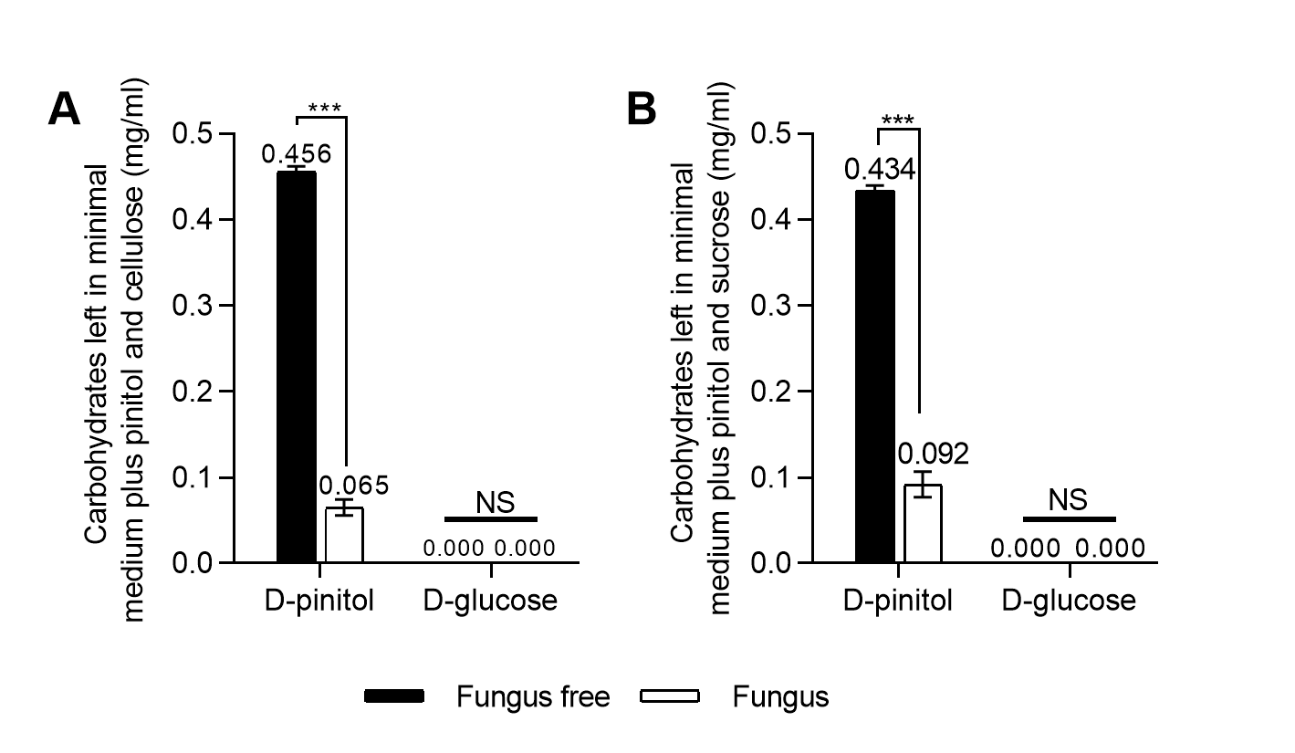


**Fig. S4 Carbohydrate composition in *L. procerum* colonized area of minimal medium with ammonium and cellulose (a) or sucrose (b) at 5th days.** Asterisk indicates significant difference between fungus and fungus free (* *P* < 0.05, ** *P* < 0.01, *** *P* < 0.001). NS means no significance.


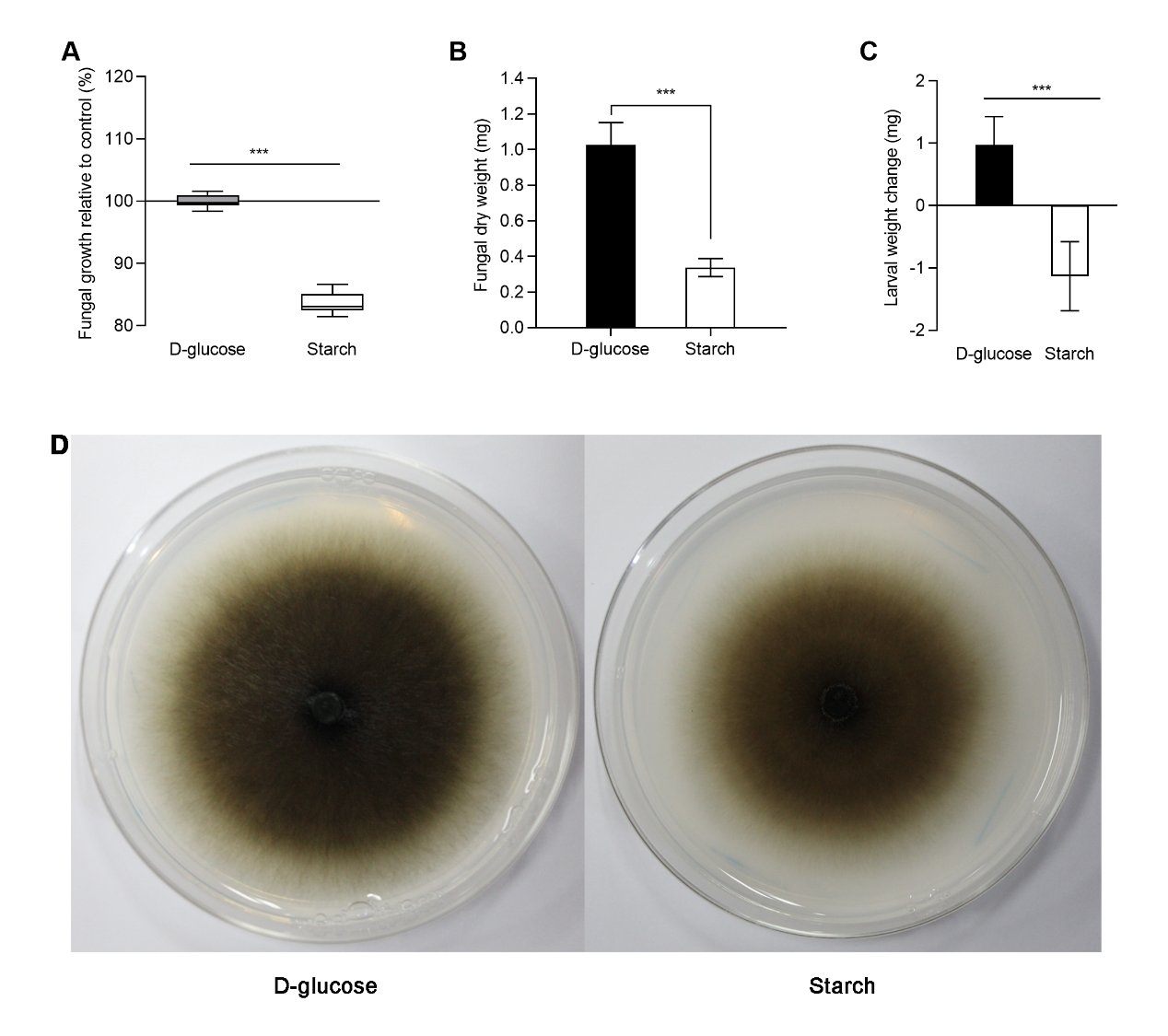


**Fig. S5 Effects of D-glucose and starch on the growth of *L. procerum*, and RTB larvae.** **a** Growth of *L. procerum*. **b** Dry weight of *L. procerum*. **c** Larval weight change. **d** Representative growth of *L. procerum* on minimal medium with D-glucose (*Left*) and starch (*Right*). Error bars of **a**, **b** and **c** represent SEs of at least 18 biological replicates. Asterisk indicates significant difference between D-glucose and starch (* *P* < 0.05, ** *P* < 0.01, *** *P* < 0.001). NS means no significance.


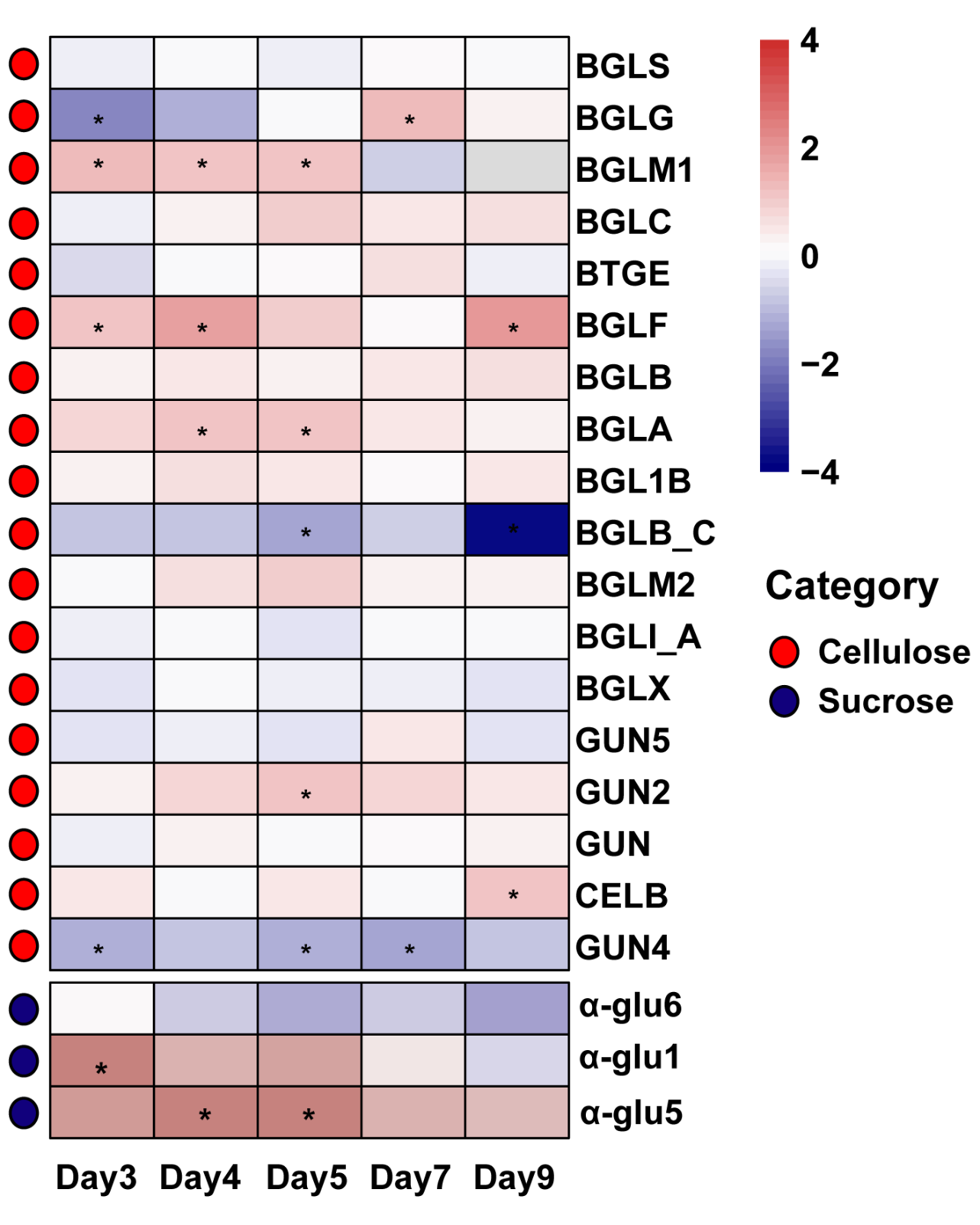


**Fig. S6 Heatmap of gluconeogenesis related genes for cellulose and sucrose.** Asterisk (*) indicates that an absolute value of log_2_Ratio ≥ 1 and FDR < 0.05.


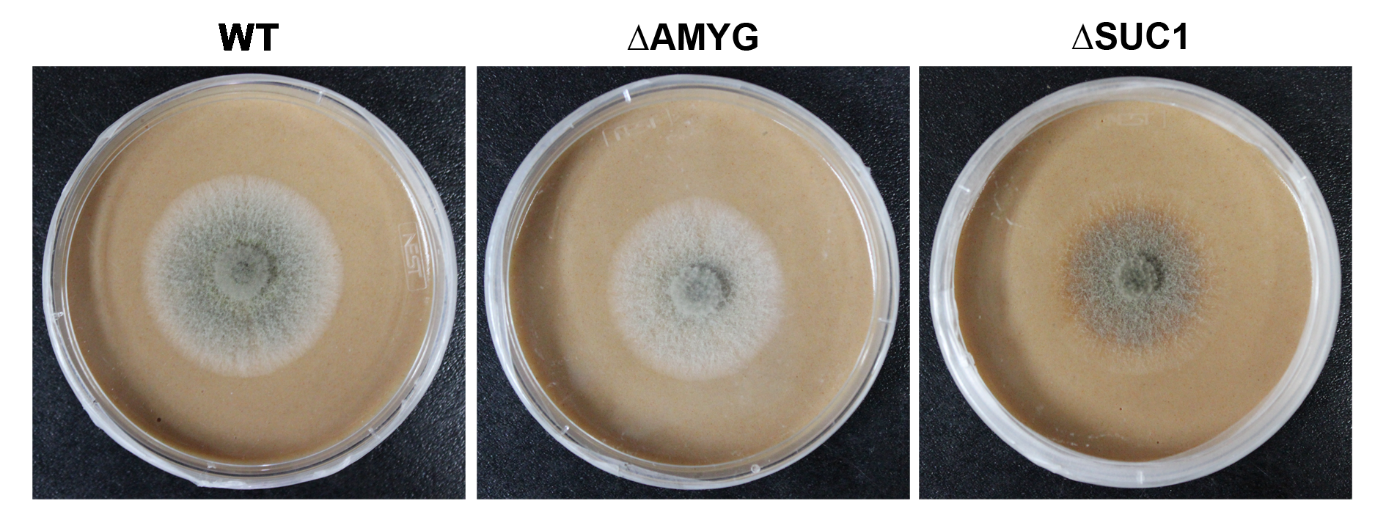


# **Fig. S7 Representative growth of WT, ΔAMYG mutant and ΔSUC1 mutant of *L. procerum* on phloem medium infused with ammonium (0.019 mol/L of NH_4_Cl).**


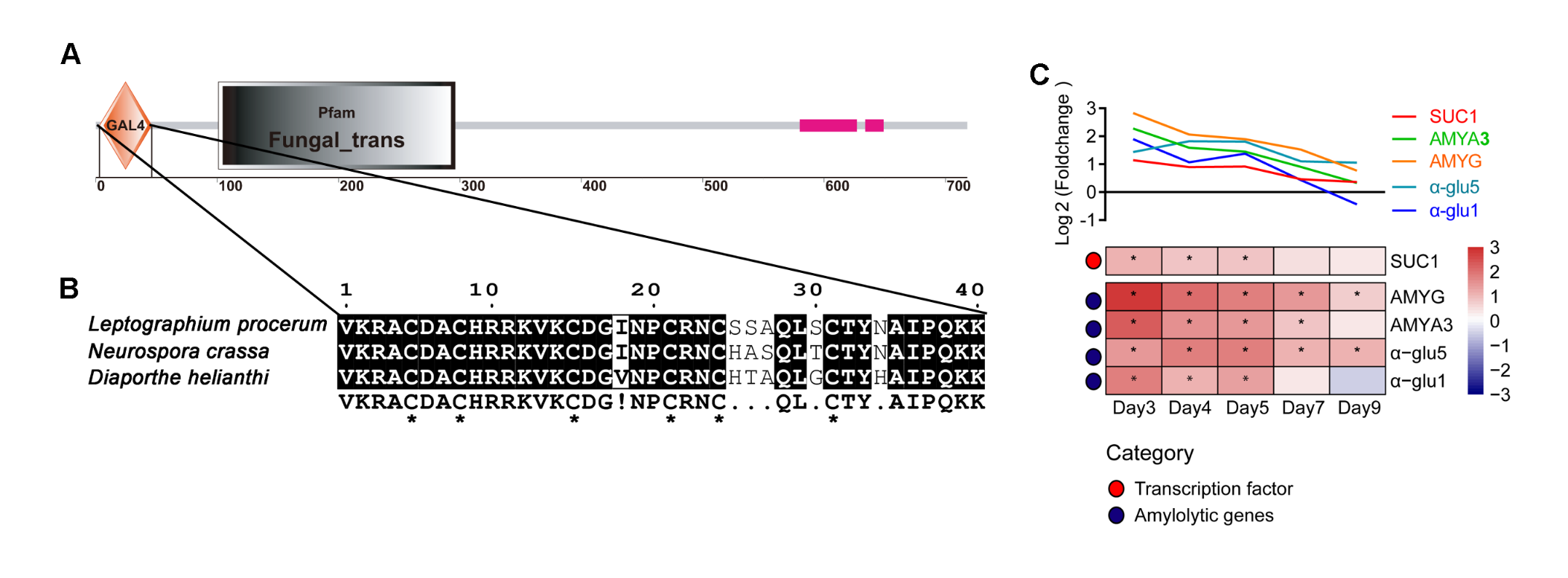


**Fig. S8 Prediction of SUC1 in *L. procerum*. a** The typical domains of SUC1. **b** Alignment of the amino acid sequences around GAL4 domain. **c** Expression trends (*Top*) and Heatmap (*Bottom*) of SUC1 and amylolytic genes at different time points. Asterisk (*) of C indicates that an absolute value of log_2_Ratio ≥ 1 and FDR < 0.05.


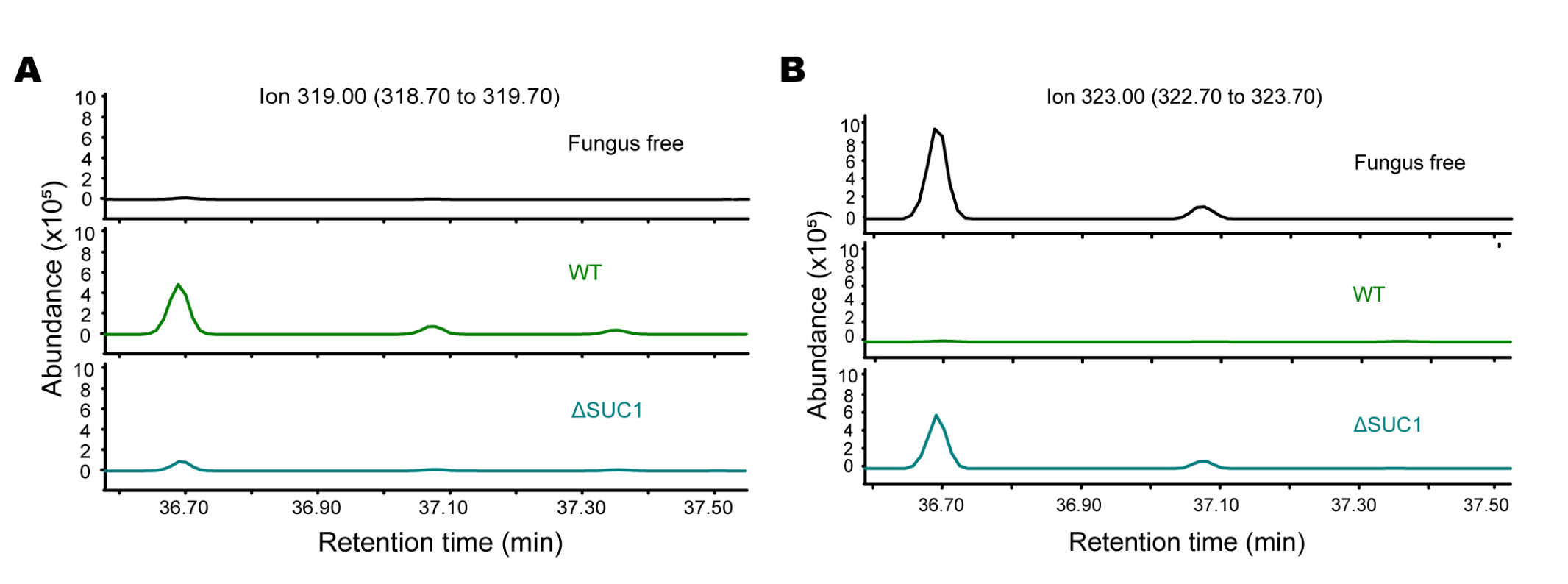


**Fig. S9 Functional verification of SUC1. a** Representative growth of WT and ΔSUC1 mutant of *L. procerum* on desugared phloem medium at day 5. **b** GC-MS traces of natural glucose (*Left*) and ^13^C_6_-labeled glucose (*Right*).


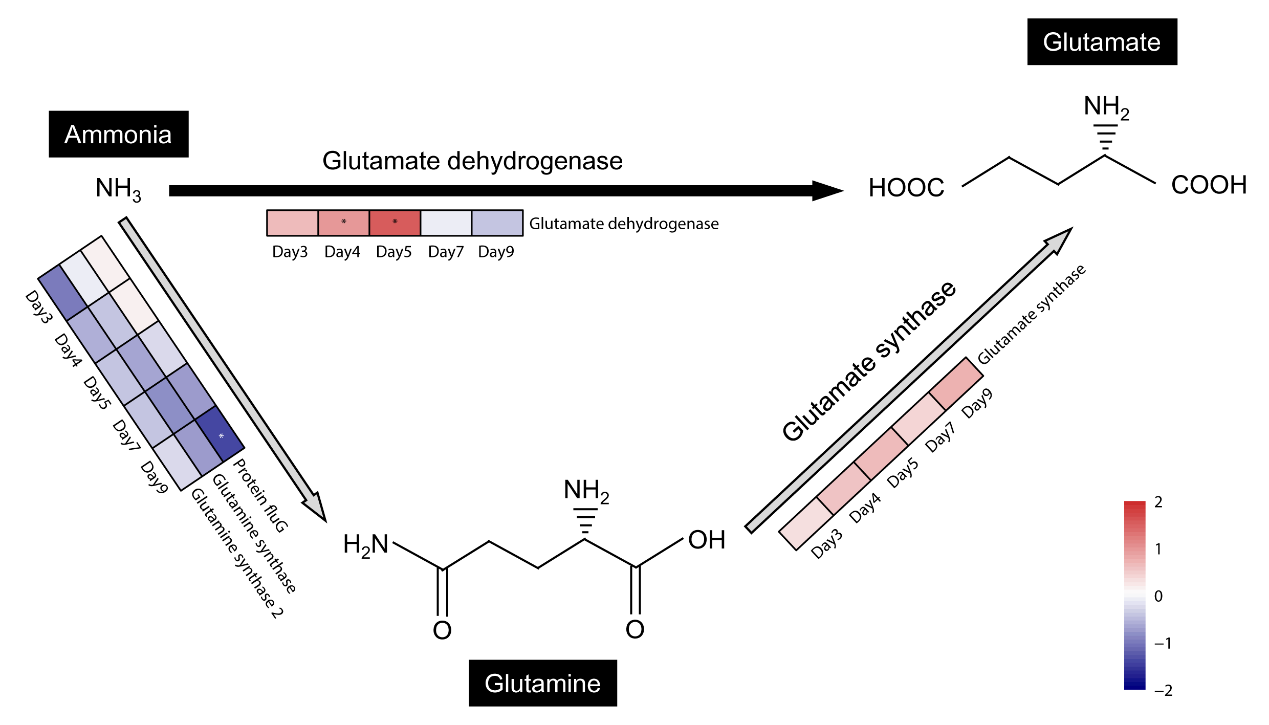


**Fig. S10 Impacts of ammonium exposure on the gene expression related to utilization of ammonia in nitrogen metabolism.** Schematic showing key genes of ammonia utilization and heatmap of these genes at different time point. Asterisk (*) indicated that an absolute value of log_2_Ratio ≥ 1 and FDR < 0.05.
